# Targeting tumor-intrinsic S100 calcium-binding protein A1 augments antitumor immunity and potentiates immunotherapy efficacy

**DOI:** 10.1038/s41392-025-02190-2

**Published:** 2025-03-17

**Authors:** Yufeng Guo, Rui Wan, Jianchun Duan, Li Yuan, Zhijie Wang, Jia Zhong, Xue Zhang, Zixiao Ma, Hua Bai, Jie Wang

**Affiliations:** 1 National Cancer Center/National Clinical Research Center for Cancer/Cancer Hospital, Chinese Academy of Medical Sciences and Peking Union Medical College, Beijing, 100021 China; 2 Department of Clinical Research, State Key Laboratory of Oncology in South China, Guangdong Key Laboratory of Nasopharyngeal Carcinoma Diagnosis and Therapy, Guangdong Provincial Clinical Research Center for Cancer, Sun Yat-sen University Cancer Center, Guangzhou, 510060 China; 3 CAMS Key Laboratory of Translational Research on Lung Cancer, State Key Laboratory of Molecular Oncology, Department of Medical Oncology, National Cancer Center/National Clinical Research Center for Cancer/Cancer Hospital, Chinese Academy of Medical Sciences and Peking Union Medical College, Beijing, 100021 China

**Keywords:** Cancer microenvironment, Tumour immunology

## Abstract

Immune checkpoint blockade (ICB) has revolutionized cancer treatment, but the therapeutic response is highly heterogeneous, which highlights the necessity for developing predictive biomarkers and overcoming ICB resistance. Cancer cell-intrinsic features, especially those that can be dynamically monitored via liquid biopsy, represent a broader scope for biomarker development. In addition, a potential mode of ICB resistance is tumor-intrinsic mechanisms leading to an immunosuppressive tumor microenvironment (TME). However, the underlying interactive network remains elusive, and the generalizable biomarkers and targeting strategies are still lacking. Here, we uncovered the potential of plasma S100 calcium-binding protein A1 (S100A1) for determining ICB efficacy via liquid biopsy of patients with lung cancer. Multiomics and functional studies have suggested that tumor-intrinsic S100A1 expression correlated with an immunologically “cold” TME and resistance to ICB in multiple syngeneic murine tumors and tissue samples from patients with lung cancer. Mechanistic investigations demonstrated that interfering with the tumor-intrinsic S100A1/ubiquitin-specific protease 7/p65/granulocyte-macrophage colony-stimulating factor (GM-CSF) modulatory axis could potentiate an inflamed TME by promoting M1-like macrophage polarization and T cell function. GM-CSF priming was sufficient to enhance the ICB response in tumors with high S100A1 expression in preclinical models. These findings define S100A1 as a potential blood-based biomarker and a novel synergistic target for cancer immunotherapy.

## Introduction

In recent years, ICB has brought about significant advancements, reshaping the treatment landscape for various cancers, including non-small cell lung cancer (NSCLC), melanoma, and breast cancer, in which immune checkpoint inhibitors (ICIs) targeting the programmed cell death protein 1 (PD-1) and its ligand (PD-L1), either as monotherapies or in combination, have become the standard regimen. These therapies focus on overcoming immune evasion mechanisms in the TME to reinvigorate T-cell-mediated antitumor immunity.^1^ The success of ICB is often linked to the presence of an “inflamed” TME, which is marked by a significant infiltration of tumor-specific CD8^+^ T cells required to trigger an effective cytotoxic immune response. However, the therapeutic response is heterogeneous, with only a small fraction of patients achieving a long-term, sustained response, and the response rates in advanced cancers typically ranging from 10 to 25%.^2^ The overall unsatisfactory response to ICIs, coupled with significant variability in individual patient outcomes, underscores the necessity for improvements in immunotherapy efficacy and the development of predictive biomarkers.

Accumulating evidence suggests that the immune-suppressive TME or immunologically “cold” TME represents a major obstacle to effective immunotherapy.^3^ This type of TME is characterized by a lack of immune effector cell infiltration, such as CD8^+^ cytotoxic T lymphocytes, type 1 helper T cells, natural killer cells, mature dendritic cells (DCs), and proinflammatory M1-like tumor-associated macrophages (TAMs). Instead, it shows an increased presence of immunosuppressive regulatory T cells (Tregs), exhausted CD8^+^ T cells, immature or tolerogenic DCs, anti-inflammatory M2-like TAMs, and myeloid-derived suppressor cells.^3^ However, the current understanding of the communication network between cancer cells and immune cells is still limited, and the factors driving these distinct immunomodulatory scenarios remain unclear.^4^ Recent study reveals an essential role of tumor-mediated immunosuppression in immune evasion and the ICB response.^5^ Tumor-intrinsic genetic changes in oncogenes, tumor suppressor genes, or DNA repair genes can modulate the immune milieu.^6^ Importantly, tumor cell-intrinsic factors also influence the communication with the immune compartment via various mechanisms such as the secretome, direct cell-to-cell contact, extracellular vesicles, and nutrient metabolism.^6^ In addition, tumor cell-intrinsic features, including PD-L1, microsatellite instability (MSI), mismatch repair (MMR) deficiency, and tumor mutational burden (TMB), are identifiable in tumor tissues and have shown potential as biomarkers to guide immunotherapy.^5^ However, clinically applicable and efficient biomarkers are lacking, and blood-based biomarkers, which can be evaluated via liquid biopsy for dynamic monitoring, are yet to be explored to overcome the limitations of tissue biopsies. These findings highlight the importance of discovering potential biomarkers and developing treatment approaches for immunologically “cold” tumors from the perspective of tumor cell-intrinsic mechanisms.

Ca^2+^ is a crucial intracellular second messenger and plays essential roles in cell proliferation, differentiation, cell death, and various cell signal transduction pathways.^7^ Ca^2+^ signaling and homeostasis and its impact on tumor formation, progression, metastasis, and the TME have attracted widespread attention.^8^ Increasing evidence suggests that tumor cells promote multiple malignant biological behaviors, such as tumor survival, proliferation, and immune evasion, by altering Ca^2+^ homeostasis.^7^ The regulation of calcium homeostasis involves various molecular regulatory mechanisms, including Ca^2+^-binding proteins, calcium pumps, and calcium channels, among which Ca^2+^-binding proteins are critical mediators in the execution of Ca^2+^-related physiological functions. Notably, S100A1 is crucial for regulating intracellular Ca^2+^ signaling and homeostasis. S100A1 contains two EF-hand type Ca^2+^-binding domains, allowing it to respond to changes in intracellular and extracellular calcium concentrations by altering its conformation and interacting with various targets, such as the ryanodine receptor, sarcoplasmic/endoplasmic reticulum calcium ATPase, and p53, to perform diverse biological functions.^9–12^ Recent studies have shown that S100A1 is aberrantly expressed in multiple types of solid tumors; is involved in biological processes such as cell cycle regulation, apoptosis, malignant invasion, and metastasis.^10^ However, the development of therapies targeting S100A1 is challenging due to its immunological and biochemical characteristics. S100A1 functions not only as an intracellular signaling molecule but also as a secreted protein, complicating therapeutic approaches. Furthermore, many of its interactive targets have only recently been discovered.^13^ The S100A1-target complexes and their resulting functions, especially in modulating the TME and antitumor immunity and in potentiating cancer immunotherapy, have not yet been completely defined.

In this study, we aimed to assess the potential of S100A1 as a biomarker and novel therapeutic target for immunotherapy. We found that the expression of S100A1 was related to the ICB response and patient survival. Functional and mechanistic investigations have characterized the role of tumor-intrinsic S100A1 ablation in promoting M1-like macrophage polarization via the S100A1/ubiquitin-specific protease 7 (USP7)/p65/GM-CSF regulatory axis, thus enhancing T-cell-mediated antitumor immunity and potentiating immunotherapy efficacy. Animal model studies suggest that S100A1 is a potential biomarker for combining GM-CSF and ICIs to treat immunologically “cold” tumors. Our results highlight the potential of S100A1 as a predictor for the response to ICB and provide a rationale for the combination of GM-CSF with ICB in tumors exhibiting elevated S100A1 expression.

## Results

### S100A1 expression is associated with immunotherapy efficacy

**Fig. 1:**
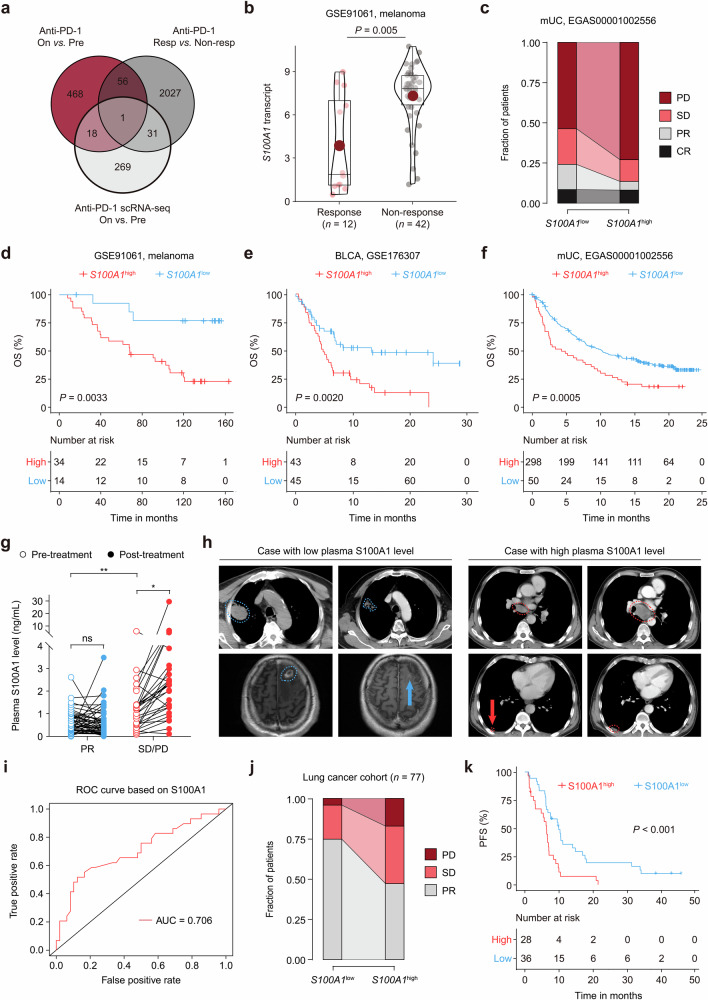
S100A1 expression is correlated with immunotherapy outcomes. **a** Venn diagram depicting the overlap profile of differentially-expressed genes between pretreatment and on-treatment samples in the GSE91061 cohort, nonresponders and responders in the GSE91061 cohort, pretreatment versus on-treatment tumor cells in the EGAS00001004809 dataset. **b** Quantitative analysis of *S100A1* transcripts in tumor samples from patients who were responsive (*n* = 12) or not responsive (*n* = 42) to ICB. **c** Percent bar chart depicting the difference in the response status between patients with high or low *S100A1* mRNA expression in the EGAS00001002556 cohort. **d**–**f** Kaplan‒Meier estimates of the OS between patients with high or low *S100A1* mRNA expression in the GSE91061 (**d**), GSE176307 (**e**), and EGAS00001002556 (**f**) cohorts. The red and black lines represent patients whose *S100A1* mRNA expression was above and below the cutoff (determined by the Youden index derived from the ROC curve). **g** Quantitative analysis of the changes in paired plasma S100A1 expression in patients in the pretreatment versus on-treatment with ICIs. **h** Radiological examination of lung cancer patients with low (left panel) or high (right panel) plasma S100A1 expression before and after receiving ICIs. **i** Logistic ROC analysis of plasma S100A1 expression in stratifying responders and nonresponders to ICB. **j** Percent bar chart depicting the difference in the response status between lung cancer patients with high or low plasma S100A1 expression. **k** Kaplan‒Meier estimates of the PFS of lung cancer patients with high or low plasma S100A1 expression. The red and black lines represent patients whose plasma S100A1 expression was above and below the cutoff (determined by the Youden index derived from the ROC curve)

While ICIs have become the standard regimen of neoadjuvant, adjuvant, and first-line treatment for multiple advanced-stage solid tumors, effective biomarkers are still lacking, and the response and resistance mechanisms remain unclear. To gain insight into the genetic landscape of tumors that are responsive or resistant to ICIs, we integrated the published bulk RNA-seq data of a melanoma immunotherapy cohort (GSE91061)^14^ and a single-cell RNA sequencing (scRNA-seq) dataset of patients with breast cancer receiving anti-PD-1 treatment (EGAS00001004809).^15^ We first compared the transcriptomes of paired pretreatment versus on-treatment samples and nonresponders versus responders in GSE91061; we also compared the transcriptomes of pretreatment versus on-treatment tumor cells in EGAS00001004809. After setting the threshold to a *p* value < 0.05 and Log_2_(fold change) > 0.25, we obtained the top 543 and 319 highly expressed genes in the on-treatment groups of the GSE91061 and EGAS00001004809 samples, respectively, and 2115 highly expressed genes in the nonresponders of the GSE91061 cohort. We examined the overlap of these comparisons and found that the expression of *S100A1* was associated with the ICB response (Fig. 1a).

We observed that tumor cell-intrinsic *S100A1* mRNA expression was induced upon ICB (Supplementary Fig. 1). Importantly, *S100A1* mRNA expression was higher in the nonresponder group than that in the responder group (Fig. 1b). The *S100A1*^low^ group had higher proportions of patients with partial response (PR; 15.71% *vs*. 5.41%) and stable disease (SD; 22.22% *vs*. 13.51%) and lower proportions of patients with progressive disease (PD; 53.64% *vs*. 72.97%) than did the *S100A1*^high^ group (Fig. 1c). Kaplan‒Meier analyses of overall survival (OS) in patients receiving ICIs revealed that the *S100A1*^low^ group achieved a longer OS than did the *S100A1*^high^ group across multiple immunotherapy cohorts, including melanoma,^14^ bladder cancer (BLCA),^16^ and metastatic urothelial carcinoma (mUC)^17^ cohorts (all log-rank *p* < 0.01) (Fig. 1d–f), suggesting the potential of S100A1 in determining immunotherapy efficacy.

Because S100 proteins can be secreted or released from cells, we wondered whether the level of secreted S100A1 might correlate with *S100A1* mRNA and protein expression. The S100A1 expression detected in the supernatant of the HEK293T cells via enzyme-linked immunosorbent assay (ELISA) was positively correlated with its mRNA and protein expression (Supplementary Fig. 2a–e). Moreover, we collected paired plasma and tissue samples from forty-four patients with lung cancer (NSCLC, SCLC, and other subtypes) at the National Cancer Center/Cancer Hospital, Chinese Academy of Medical Sciences, from December 2015 to February 2023 (Data S1). As shown by immunohistochemistry (IHC) testing (Supplementary Fig. 2f) and ELISA, the plasma S100A1 levels were positively correlated with tissue S100A1 expression (Supplementary Fig. 2g). These results enabled us to evaluate the predictive value of plasma S100A1 levels, which can be noninvasively detected in patient blood samples. Seventy-seven blood samples from patients with lung cancer (NSCLC, SCLC, and other subtypes) who received ICIs were collected (Data S2). The plasma S100A1 levels were higher in patients with SD/PD compared to those with PR (Fig. 1g). No notable difference was found between pretreatment and post-treatment plasma S100A1 levels in the patients with PR. However, the post-treatment plasma S100A1 level was greater than the pretreatment level in the patients with SD/PD (Fig. 1g). Radiological examination revealed a difference in the clinical response between the two groups: Baseline images of the patient with a low plasma S100A1 level displayed a lesion in the upper lobe of the right lung (4.6 cm × 3.6 cm; blue outline) based on computed tomography (CT) scan and a metastatic nodule in the left frontal lobe (1.8 cm × 1.4 cm; blue outline) detected via magnetic resonance imaging. After four cycles of chemotherapy combined with ICB, the right lung mass was reduced in size (3.6 cm × 2.6 cm; blue outline), whereas the left frontal lobe metastasis became unmeasurable with a notable reduction in surrounding edema (blue arrow) following six cycles of treatment (Fig. 1h), which was classified as a PR based on the modified Response Evaluation Criteria in Solid Tumors (RECIST 1.1).^18^ For the patient with a high plasma S100A1 level, a CT scan revealed a subpleural nodule in the lower lobe of the right lung (1.4 cm × 0.8 cm; red outline) and a lesion in the mediastinal lymph node (short axis: 1.9 cm; red outline and arrow). After three cycles of ICB treatment, a follow-up CT scan revealed a slight increase in the size of the right lung nodule (2.1 cm × 1.0 cm; red outline) and enlargement of the mediastinal lymph node (short axis: 3.6 cm; red outline) (Fig. 1h). According to RECIST 1.1, the patient’s response to ICB was classified as PD. The plasma S100A1 expression had an area under the curve (AUC) of 0.71 in predicting the response to immunotherapy (Fig. 1i). Notably, of the 43 S100A1^low^ patients, 32 (74.4%) had PR, 9 (20.9%) had SD, and 2 (4.7%) had PD. In contrast, 16 (47.1%) of 34 patients in the S100A1^high^ group achieved PR, 12 (35.3%) had SD, and 6 (17.6%) had PD (Fig. 1j). Besides, no statistically significant differences in age, gender, smoking history, histology, tumor stage, differentiation, driver mutation status, treatment, or lines of treatment were observed between the groups (Supplementary Table 1). Furthermore, the S100A1^low^ group exhibited a longer progression-free survival (PFS) compared to the S100A1^high^ group (Fig. 1k). These findings imply that plasma S100A1 levels could serve as a potential biomarker for patients treated with immunotherapy and that S100A1 might be involved in antitumor immunity.

### S100A1 regulates tumor immune evasion and the response to immunotherapy

**Fig. 2:**
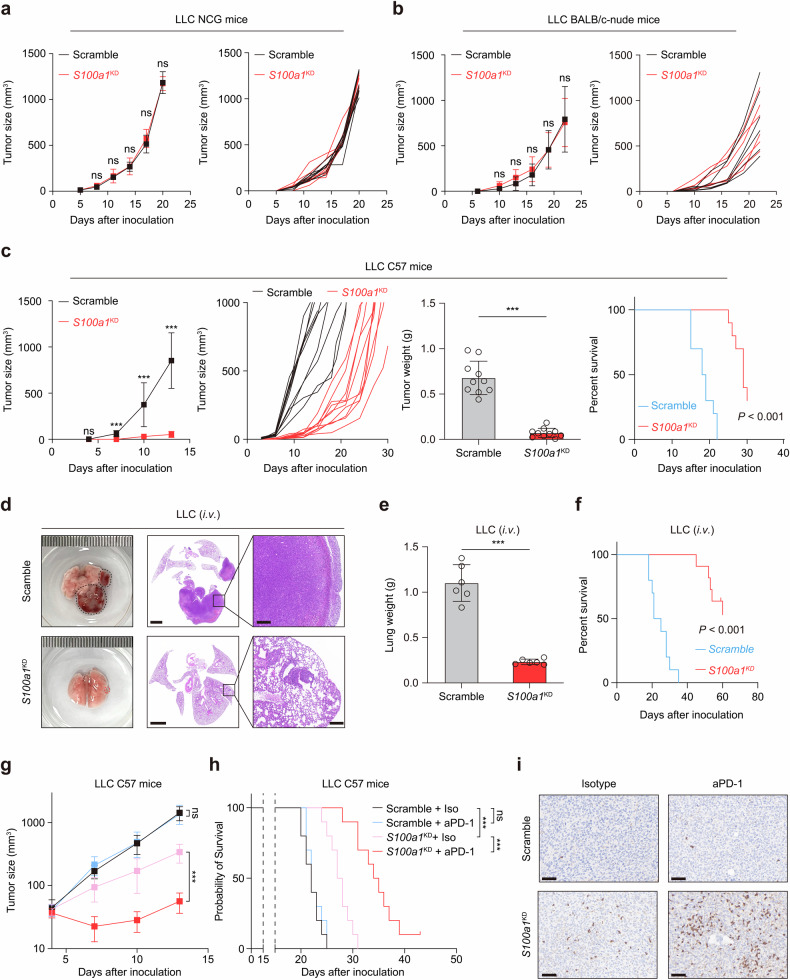
Tumor-intrinsic S100A1 affects tumor immunity and the sensitivity to ICB. **a**, **b** Tumor growth curves of control and *S100a1*^KD^ LLC in NCG mice (**a**) and nude mice (**b**). **c** Tumor growth curves, tumor weight, and survival analysis of control and *S100a1*^KD^ LLC in C57BL/6J mice. **d** Representative images of HE-stained lungs from the orthotopic tumor models receiving intravenous injection of control (upper panel) or *S100a1*^KD^ LLC cells (lower panel). The scale bars represent 2000 μm and 200 μm, respectively. **e**, **f** Lung weights (**e**) and survival analysis (**f**) of mice receiving intravenous injection of control or *S100a1*^KD^ LLC cells. **g**, **h** Tumor growth curves (**g**) and survival analysis (**h**) of control or *S100a1*^KD^ LLC tumor-bearing mice receiving anti-PD-1 or isotype. **i** Representative images of the IHC analysis of CD8 protein expression in tumors from different treatment groups as described in (**g**). The scale bars represent 100 μm

To analyze S100A1 function, we used the LLC cell line, considered immunologically “cold” and resistant to immunotherapy,^19,20^ to generate an endogenous *S100a1*-knockdown model. The stable knockdown of *S100a1* using shRNA was verified through quantitative polymerase chain reaction (qPCR; Supplementary Fig. 3a) and immunoblotting (Supplementary Fig. 3b). Upon *S100a1* loss in LLC cells, the supernatant S100A1 level also decreased (Supplementary Fig. 3c). No significant changes in in vitro cell proliferation were observed upon *S100a1* ablation (Supplementary Fig. 3d). To determine the role of S100A1 in vivo, we inoculated LLC cells transfected with the *S100a1* shRNAs (*S100a1*^KD^) or the scramble control subcutaneously into NOD-*Prkdc*^*em26Cd52*^*Il2rg*^*em26Cd22*^/NjuCrl (NCG) triple-immunodeficient mice and observed no significant differences (Fig. 2a), indicating that S100A1 was not essential for cancer cell-intrinsic fitness. Moreover, reducing S100A1 expression also did not affect tumor progression in immune-deficient BALB/c-nude mice (Fig. 2b). Notably, *S100a1* loss delayed LLC tumor growth and prolonged survival in immune-competent C57BL/6J mice (Fig. 2c), suggesting that T-cell-mediated immunity contributed to the antitumor activity of tumor-intrinsic *S100a1* loss. CT26 is a murine model of colon cancer with low MSI and inherited immunotherapy resistance.^21^ 4T1 is a triple-negative breast cancer (TNBC) model known for its low immunogenicity and resistance to ICB.^22^ We obtained similar results with the CT26 and 4T1 subcutaneous syngeneic tumor models (Supplementary Fig. 4a, b). We also constructed a mouse orthotopic model via intravenous injection of LLC cells. Knockdown of S100A1 inhibited LLC tumor development (Fig. 2d), with the lung weight decreasing by approximately 75% (Fig. 2e) and mouse survival prolonged (Fig. 2f). These findings suggest that ablation of S100A1 suppresses tumor growth across multiple tumor models and mark tumor-intrinsic S100A1 as a potential target to boost antitumor immunity in various solid tumors. To rule out the possibility that tumor-intrinsic *S100A1* might contribute to the implantation of tumors, we used a doxycycline (Dox)-inducible system to abrogate *S100A1* expression in LLC model (Supplementary Fig. 4c, d) and found that Dox-induced *S100a1* loss attenuated the growth of established tumors and extended the survival of tumor-bearing mice (Supplementary Fig. 4e), similar to the results of the tumors transfected with the constitutive *S100a1* shRNA. Moreover, combining S100A1 depletion with anti-PD-1 therapy effectively attenuated LLC tumor growth (Fig. 2g, h) and increased CD8^+^ T-cell infiltration (Fig. 2i). These observations suggest a potent T cell presence following the treatment of *S100a1*^KD^ tumors with ICB and imply a role of S100A1 in remodeling the TME.

### Ablation of tumor-intrinsic S100A1 reverses the immunosuppressive TME

**Fig. 3:**
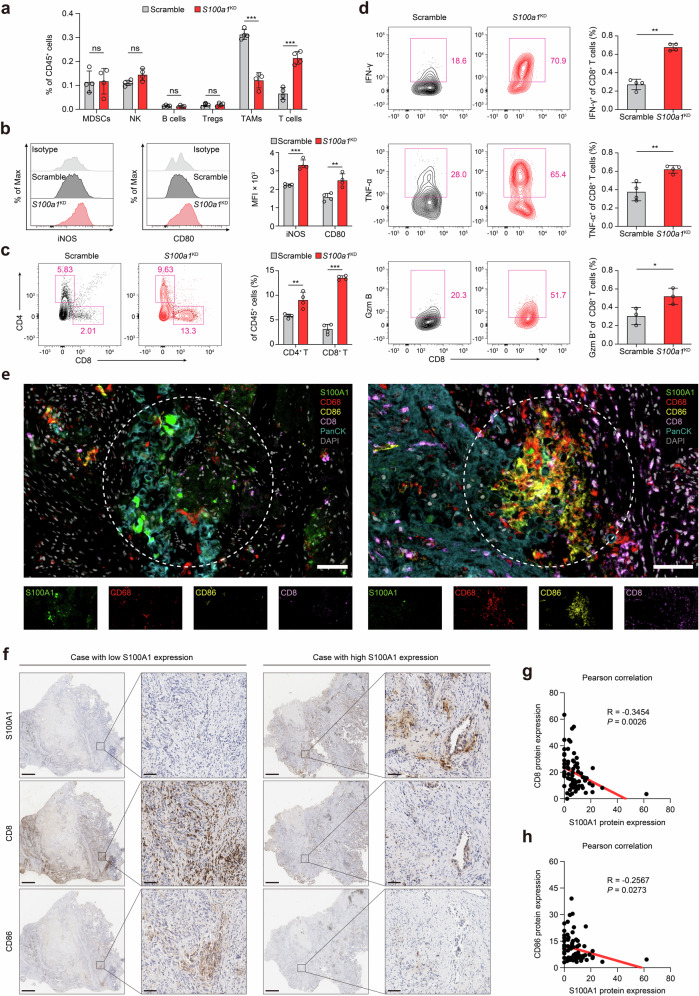
Tumor-intrinsic S100A1 is associated with the immunosuppressive TME. **a** Flow cytometry analysis of different immune cell populations in the TIME upon *S100a1* knockdown. **b** iNOS^+^ and CD80^+^ macrophages determined by flow cytometry. The data are presented as the means ± SEMs (*n* = 4, **p < 0.01, ***p < 0.001). **c** CD4^+^ and CD8^+^ T cells determined by flow cytometry. The data are presented as the means ± SEMs (*n* = 4, **p < 0.01, ***p < 0.001). **d** IFN-γ^+^, TNF-α^+^, and GZMB^+^ T cells determined by flow cytometry. The data are presented as the means ± SEMs (*n* = 3‒4, *p < 0.05, **p < 0.01). **e** Multiplexed immunofluorescence images showing the heterogeneous TIME between S100A1-high- and S100A1-low-expressing tumors in NSCLC patient samples via antibodies against S100A1, CD68, CD86, CD8, and panCK, as well as DAPI staining. The scale bar represents 50 μm. **f** Representative images of the IHC analysis of CD8 and CD86 protein expression in S100A1-high- and S100A1-low-expressing tumors from NSCLC patient samples. The scale bars represent 2000 μm and 200 μm, respectively. **g**, **h** Pearson correlation analyses of S100A1 protein expression with CD8 (**g**) and CD86 (**h**) protein expression in NSCLC patient samples

Considering S100A1’s involvement in immune suppression and tumor progression, we explored whether S100A1 depletion influences the tumor immune microenvironment (TIME) via flow cytometry. Since the clinical cohort examining the relationship between plasma S100A1 expression and immunotherapy outcome was comprised of samples mainly from patients with lung cancer and LLC is characterized by an immunosuppressive TME and resistance to immunotherapy,^20^ we selected the LLC lung cancer model to further investigate the phenotypic effect of *S100a1* loss. Ex vivo analysis of *S100a1*^KD^ tumors showed substantially decreased recruitment of TAMs, along with an increase in both the percentage and number of T cells within the TIME (Fig. 3a and Supplementary Fig. 5a). The macrophage phenotype analysis indicated that markers of M1-like polarization [inducible nitric oxide synthase (iNOS), CD80, CD86, and I-A/I-E] were more prevalent in *S100a1*^KD^ tumors (Fig. 3b and Supplementary Fig. 5b). *S100a1*^KD^ tumors exhibited increased infiltration of CD8^+^ and CD4^+^ T cells than control tumors did (Fig. 3c and Supplementary Fig. 5c). Tumor cell-intrinsic *S100a1* ablation promoted CD8^+^ T-cell activation and proliferation (Supplementary Fig. 5d, e). However, the percentage of PD-1^+^ TIM-3^+^ exhausted CD8^+^ T cells was comparable between the control and *S100a1*^KD^ LLC tumors (Supplementary Fig. 5f). In addition, the tumor-infiltrating CD8^+^ T cells within the *S100a1*^KD^ tumors demonstrated enhanced effector activity compared to those in the scramble control, as shown by their ability to secrete interferon-γ (IFN-γ), granzyme B (GZMB) and tumor necrosis factor-α (TNF-α) (Fig. 3d). The increased levels of M1-type TAM and CD8^+^ T infiltration and the low level of S100A1 expression were confirmed by multiplex immunofluorescence staining of tissue samples from NSCLC patients (Fig. 3e). We next performed IHC analysis of tissue samples from seventy-four NSCLC patients (Fig. 3f and Data S3). The S100A1 protein expression showed an inverse correlation with the protein expression of CD8 and CD86 (Fig. 3g, h). These findings indicate that tumors with high S100A1 expression exhibited an immunologically “cold” TME characterized by a lack of T cell and proinflammatory M1 macrophage infiltration and that tumor-intrinsic S100A1 loss can remodel the TME to become more immunoactive.

### S100A1 knockdown promotes M1-like macrophage polarization and T-cell-mediated antitumor immunity

To assess the effect of S100A1 ablation on the TIME, we analyzed the TIME via scRNA-seq. A total of eight distinct cell subsets were identified through uniform manifold approximation and projection (UMAP), among which TAMs and T cells exhibited prominent altered infiltration between tumors from *S100a1*^KD^ mice and control mice (Supplementary Fig. 6a–c), which aligned with our previous observations. To further elucidate how *S100a1*^KD^ affects the TAMs transcriptome, we conducted an unbiased secondary clustering analysis of these cells, revealing seven distinct subpopulations (Supplementary Fig. 6d). The cells in C0_TAM-*Spp1* presented exhibited elevated expression of *Arg1*, *Thbs1*, *Pdpn*, *Adam8*, and *Abca1*, a pattern that aligns with the characteristics of immune-suppressive M2-like macrophages (Supplementary Fig. 6e). The cells in C1_TAM-*C1q* were marked by elevated expression of major histocompatibility complex (MHC)-II genes (*H2.DMb1*, *H2.DMa*, *H2.Eb1*, *H2.Aa*) and *Aif1*, resembling classic inflammatory M1-like macrophages (Supplementary Fig. 6f). The cells in C6_Mac-*Pparg* presented high *Plet1*, *Ear2*, *Krt79*, *Cidec*, and *Ear1* expression, which was consistent with a classic tissue-resident alveolar macrophage phenotype (Supplementary Fig. 6g). *S100a1*^KD^ markedly expanded the C0_TAM-*Spp1*, C1_TAM-*C1q*, and C6_Mac-*Pparg* populations (Supplementary Fig. 6d). The Spp1^+^ TAMs and C1q^+^ TAMs were previously reported to exhibit both “classically activated” (M1) and “alternatively activated” (M2) phenotypes,^23^ indicating that *S100a1*^KD^ could reprogram TAMs into the M1-like phenotype. Notably, tumor-intrinsic *S100a1*^KD^ did not affect the expression patterns of genes associated with angiogenesis or phagocytosis, both of which are essential functions of TAMs (Supplementary Fig. 6h). However, the M1-like TAM signature was uniformly increased upon *S100a1*^KD^ across all the macrophage subsets, with no comparable changes in the M2-like signature (Supplementary Fig. 6h). The inflammatory response and TNF-α signaling via NF-κβ, which is representative of proinflammatory M1-like signaling, were induced in C0_TAM-*Spp1*, C1_TAM-*C1q*, and C6_Mac-*Pparg* (Supplementary Fig. 7).

To explore how *S100a1*^KD^ affects the transcriptome of tumor-infiltrating CD8^+^ T cells, we conducted an unbiased secondary clustering of the overall T-cell population, which revealed six unique subpopulations (Supplementary Fig. 8a). The CD8^+^ Tn cells exhibited elevated expression of *Lef1* and *Tcf1* (Supplementary Fig. 8b), suggesting the naive T-cell phenotype. *S100a1*^KD^ increased the number of CD8^+^ Tn cells (Supplementary Fig. 8a). CD8^+^ Teff and CD8^+^ Tpex cells showed high expression of cytotoxic markers such as *Nkg7* and *Ccl5* (Supplementary Fig. 8c), resembling the effector CD8^+^ T cells. *S100a1*^KD^ increased the CD8^+^ Teff and CD8^+^ Tpex populations (Supplementary Fig. 8a) and promoted the expression of *Nkg7* and *Ccl5* (Supplementary Fig. 8d). The expression profile of the CD8^+^ proliferating cluster resembled that of the CD8^+^ Teff and CD8^+^ Tpex clusters, but it was marked by high *Mki67* expression (Supplementary Fig. 8c), indicating a population of highly proliferative effector CD8^+^ T cells. *S100a1*^KD^ increased the CD8^+^ proliferating population (Supplementary Fig. 8a), suggesting that *S100a1*^KD^ triggered the expansion of effector CD8^+^ T cells. Indeed, the potential of heat diffusion for affinity-based trajectory embedding (PHATE) analysis revealed the functional dynamics of tumor-infiltrating CD8^+^ T cells, progressing from a naive state to activation, acquiring effector functions, and eventually entering a proliferative state. *S100a1*^KD^ promoted the activation and proliferation of CD8^+^ T cells compared to the scramble control, which was similar to the flow cytometry results (Supplementary Fig. 8e) and suggested that tumor-intrinsic S100A1 may be involved in modulating T cell-mediated antitumor immunity. Next, we scored each CD8^+^ T-cell subset on the basis of activation, surface receptor signaling, and cytokine production levels. The contour plots showed that the effector CD8^+^ T cells (CD8^+^ Teff and CD8^+^ Tpex) presented greater antitumor capacity in *S100a1*^KD^ tumors than did their scramble counterparts (Supplementary Fig. 8f), suggesting that T cells in the *S100a1*^KD^ TME are more immune-activated.

Spatial transcriptomic analysis also revealed that *S100a1*^KD^ promoted T-cell infiltration, especially in the tumor core region (Supplementary Fig. 9a). Compared with those in the control tumors, functional annotation analysis revealed M1-like macrophage polarization in the core region of the *S100a1*^KD^ tumors (Supplementary Fig. 9b). Meanwhile, the T-cell-inflamed signature was enriched in the *S100a1*^KD^ tumors (Supplementary Fig. 9b), suggesting that *S100a1*^KD^ induces a potentiated or inflamed TME. Thus, tumor-intrinsic *S100a1* loss reshapes the TME from an immune-suppressive to an immune-active state. These findings indicate that *S100a1*^KD^ inhibits tumor progression by fostering inflammatory M1-like macrophage polarization and enhancing T-cell-mediated antitumor immunity.

### Tumor-intrinsic S100A1 modulates TAMs via GM-CSF

**Fig. 4:**
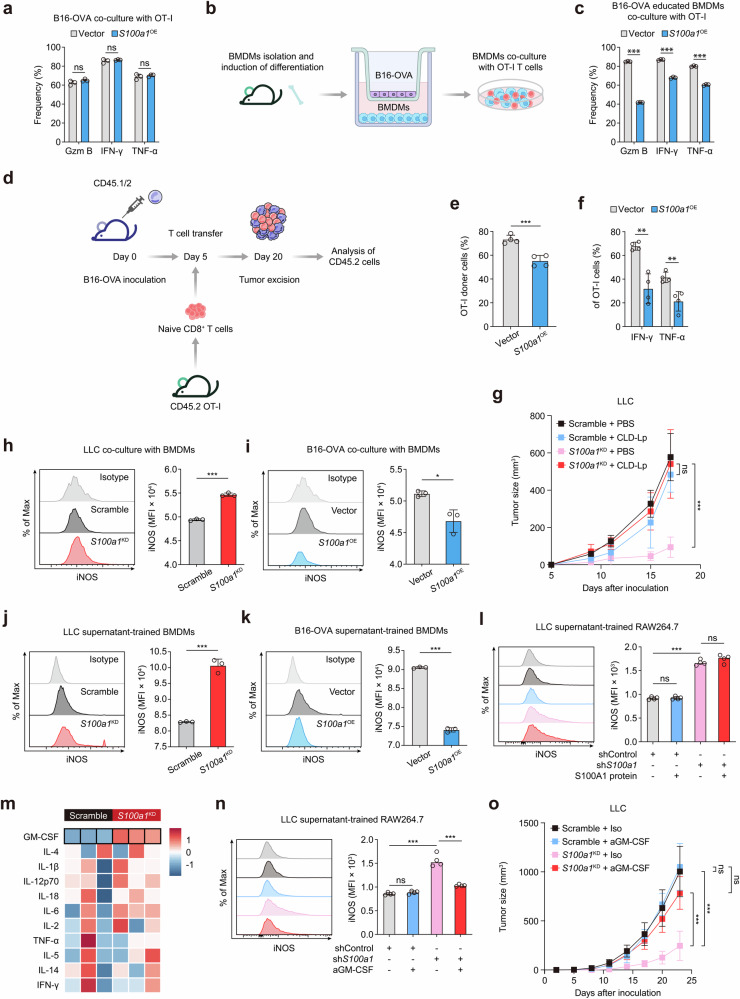
GM-CSF mediates the modulatory effect of tumor-intrinsic S100A1 on M1-like macrophage polarization. **a** Flow cytometry analysis of IFN-γ^+^, TNF-α^+^, and GZMB^+^ TCR_OT1_ after in vitro coculture with control or *S100a1*^OE^ B16-OVA cells. The data are presented as the means ± SEMs (*n* = 3, ns: not significant). **b** Schematics depicting the experimental design of in vitro coculture system of B16-OVA cells, BMDMs, and TCR_OT1_. The elements were sourced from Scidraw.io. **c** Flow cytometry analysis of IFN-γ^+^, TNF-α^+^, and GZMB^+^ TCR_OT1_ in the in vitro coculture system as described in (**b**). The data are presented as the means ± SEMs (*n* = 3, ***p < 0.001). **d** Experimental schemes showing the adoptive T-cell transfer regimen. The elements were sourced from Scidraw.io. **e**, **f** Flow cytometry analysis of TCR_OT1_ (**e**), IFN-γ^+^ TCR_OT1_, and TNF-α^+^ TCR_OT1_ (**f**) in response to the adoptive T-cell transfer regimen as described in (**d**). The data are presented as the means ± SEMs (*n* = 4, ***p < 0.001, **p < 0.01). **g** Tumor growth curves of the control or *S100a1*^KD^ LLC tumor-bearing mice receiving CLD-Lp or PBS. **h** Flow cytometry analysis of iNOS^+^ BMDMs cocultured with control or *S100a1*^KD^ LLC cells in vitro. The data are presented as the means ± SEMs (*n* = 3, ***p < 0.001). **i** Flow cytometry analysis of iNOS^+^ BMDMs cocultured with control or *S100a1*^OE^ B16-OVA cells in vitro. The data are presented as the means ± SEMs (*n* = 3, *p < 0.05). **j** Flow cytometry analysis of iNOS^+^ BMDMs treated with conditioned medium derived from control or *S100a1*^KD^ LLC cells. The data are presented as the means ± SEMs (*n* = 3, ***p < 0.001). **k** Flow cytometry analysis of iNOS^+^ BMDMs treated with conditioned medium derived from control or *S100a1*^OE^ B16-OVA cells. The data are presented as the means ± SEMs (*n* = 3, ***p < 0.001). **l** Flow cytometry analysis of iNOS^+^ BMDMs treated with conditioned medium derived from control or *S100a1*^KD^ LLC cells in the absence or presence of recombinant S100A1 protein. The data are presented as the means ± SEMs (*n* = 4, ***p < 0.001). **m** Heatmap showing cytokine profiles of supernatants from control and *S100a1*^KD^ LLC cells based on Luminex-based multiplex assays. **n** Flow cytometry analysis of iNOS^+^ BMDMs treated with conditioned medium derived from co**n**trol or *S100a1*^KD^ LLC cells in the absence or presence of anti-GM-CSF. The data are presented as the means ± SEMs (*n* = 4, ***p < 0.001). **o** Tumor growth curves of control or *S100a1*^KD^ LLC tumor-bearing mice receiving anti-GM-CSF or isotype

To further elucidate the interactive network of tumor-intrinsic S100A1, M1-like macrophage polarization, and T-cell function, we modified B16 melanoma cells to express the ovalbumin-derived CD8^+^ T-cell epitope OVA_257-264_ (SIINFEKL). This epitope is presented on the MHC class I molecule H-2Kb and is specifically recognized by T-cell receptor (TCR) transgenic OT-I CD8^+^ T cells (TCR_OT1_).^24^ Then, we directly cocultured *S100a1*^OE^ B16-OVA melanoma cells with TCR_OT1_ to determine whether tumor-intrinsic S100A1 could directly affect T-cell function. The effector function of TCR_OT1_ was not significantly changed upon coculture with parental or S100A1-expressing B16-OVA cells, as demonstrated by the secretion of IFN-γ, GZMB, and TNF-α (Fig. 4a). However, in the presence of bone marrow-derived macrophages (BMDMs) preconditioned with *S100a1*^OE^ B16-OVA cells (Fig. 4b), the effector function of TCR_OT1_ was appreciably compromised (Fig. 4c). We further examined the effects of tumor-intrinsic S100A1 on tumor-specific CD8^+^ T-cell responses in vivo. CD45.1/CD45.2 mice were inoculated with control or *S100a1*^OE^ B16-OVA cells and then subjected to adoptive transfer of CD45.2^+^ TCR_OT1_ (Fig. 4d). Compared with the tumors derived from parental cells, the *S100a1*^OE^ B16-OVA tumors had lower infiltration of adoptively transferred TCR_OT1_ (Fig. 4e and Supplementary Fig. 10a, b). Moreover, the effector function of TCR_OT1_ was attenuated in the *S100a1*^OE^ B16-OVA tumors compared with the parental cell-derived tumors (Fig. 4f and Supplementary Fig. 10c). These results suggest that tumor-intrinsic S100A1 preferentially suppresses the infiltration of tumor-specific CD8^+^ T cells and promotes T-cell dysfunction by shifting macrophage polarization toward an immunosuppressive phenotype. Liposomes encapsulating the bisphosphonate clodronate allow specific targeting of macrophages.^25^ Indeed, the depletion of macrophages via clodronate liposome (CLD-Lp) administration was sufficient to abolish the inhibitory effect of *S100a1*^KD^ on the tumor growth of LLC cells (Fig. 4g), further confirming that tumor-intrinsic S100A1 requires macrophages to manipulate T-cell immunity and thus tumorigenesis. This observation is similar to previous evidence that M1 macrophages enhance T-cell activation in cancer and improve the response to ICB.^26^ To dissect the role of tumor-intrinsic S100A1 in macrophage polarization, we employed a coculture system to examine the interaction between tumor cells and macrophages. Our results showed that coculture of BMDMs with *S100a1*^KD^ LLC cells increased the expression of the M1 marker iNOS (Fig. 4h), whereas coculture of BMDMs with *S100a1*^OE^ B16-OVA cells decreased iNOS expression (Fig. 4i). Similar outcomes were observed when RAW264.7 cells were cocultured with *S100a1*^KD^ LLC cells (Supplementary Fig. 10d). Notably, when BMDMs were conditioned with the supernatant of *S100a1*^KD^ LLC cells or *S100a1*^OE^ B16-OVA cells, iNOS expression was also induced or attenuated (Fig. 4j, k), and this effect was comparable to that of the coculture system. However, when we added purified S100A1 protein to the supernatant, the ability of the supernatant of *S100a1*^KD^ LLC cells to promote M1-like macrophage polarization was not inhibited (Fig. 4l), suggesting that *S100a1*^KD^ triggers the release of chemoattractants or modulators for immune cells, which subsequently act in a paracrine fashion to recruit and polarize macrophages toward an antitumor phenotype.

To explore the mechanism through which tumor-intrinsic *S100a1*^KD^ influences macrophage polarization, we analyzed the cytokine profiles of supernatants from control and *S100a1*^KD^ LLC cells via Luminex-based multiplex assays. The results revealed that GM-CSF levels were elevated in the supernatants of *S100a1*^KD^ LLC cells compared to controls (Fig. 4m). GM-CSF (encoded by the *Csf2 gene*) drives the development, maturation, and differentiation of myeloid cells^27^ and is an inflammatory cytokine involved in the TNF-α signaling pathway that facilitates the differentiation of monocytes into immunostimulatory M1 macrophages.^28^ In addition, GM-CSF has the potential to convert immunosuppressive M2-like TAMs into an antitumor M1-like phenotype.^27^ qPCR, immunoblotting, and ELISA confirmed that GM-CSF expression and secretion were increased in *S100a1*^KD^ LLC cells and *S100a1*^KD^ CT26 cells compared with their counterparts. In contrast, GM-CSF expression and secretion were lower in *S100a1*^OE^ MC38 cells than in their counterparts (Supplementary Fig. 11a–c). We next investigated whether tumor-derived GM-CSF mediates the effect of *S100a1*^KD^ on the M1 polarization of macrophages. The addition of a GM-CSF neutralizing antibody to the supernatant of *S100a1*^KD^ LLC cells attenuated the induction of M1-like polarization by tumor-intrinsic *S100a1*^KD^, as demonstrated by the change in iNOS expression (Fig. 4n). Notably, the GM-CSF neutralizing antibody diminished the tumor growth inhibitory effect induced by *S100a1*^KD^ (Fig. 4o). On the basis of the above results, we hypothesized that tumor-derived GM-CSF was the critical mediator of the *S100a1*^KD^-promoted inflammatory phenotype of macrophages.

### The S100A1/USP7/p65 axis transcriptionally regulates GM-CSF expression

**Fig. 5:**
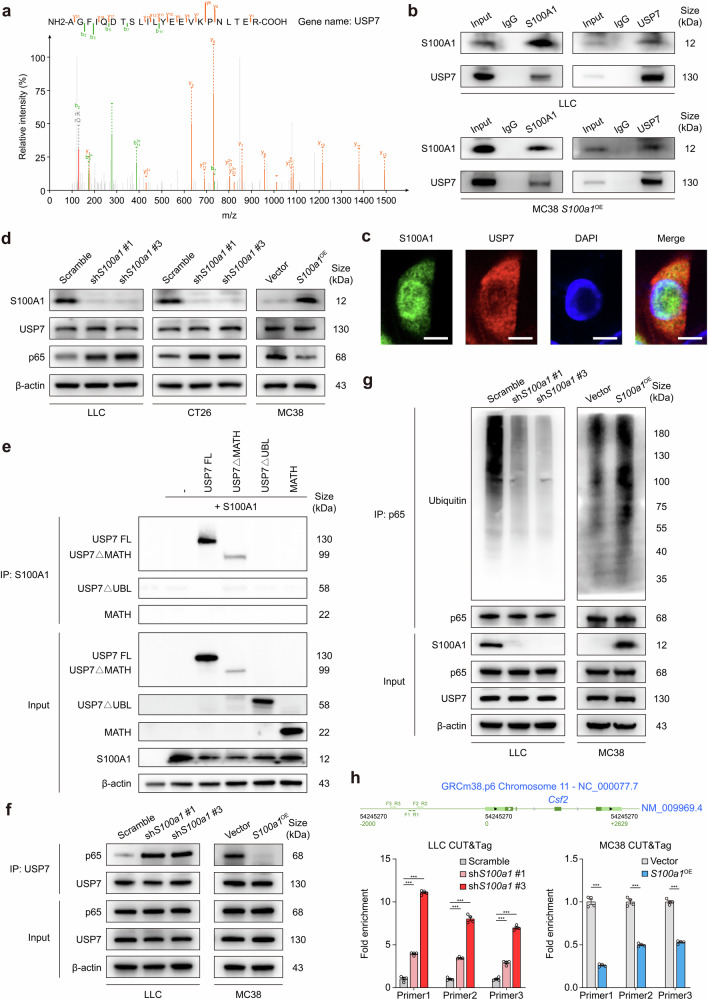
S100A1 regulates GM-CSF expression via the USP7/p65 pathway. **a** The peptide spectrum of USP7 determined by LC-MS/MS assay in the immunoprecipitates of S100A1. **b** CoIP showing the interaction of S100A1 with USP7 in LLC and *S100a1*^OE^ MC38 cells. **c** Representative immunofluorescence images showing the colocalization of S100A1 with USP7 in *S100a1*^OE^ MC38 cells. The scale bars represent 10 μm. **d** Immunoblot analysis of S100A1, USP7, and p65 protein expression in LCC and CT26 cells transfected with the *S100a1* shRNAs compared with that in cells transfected with the scramble control and in MC38 cells transfected with the *S100a1* ORF compared with that in cells transfected with the empty vector. β-actin was used as the loading control. **e** HEK293T cells were transfected with S100A1 and full-length USP7 (USP7 FL) and USP7 deletion mutants as indicated. Equal amounts of protein were immunoprecipitated with an antibody against S100A1 and immunoblotted for USP7. **f** CoIP showing the interaction of USP7 with p65 in LCC cells transfected with the *S100a1* shRNAs compared with that in cells transfected with the scramble control and in MC38 cells transfected with the *S100a1* ORF compared with that in cells transfected with the empty vector. **g** Immunoblot analysis of the ubiquitination levels of p65 in LCC cells transfected with the *S100a1* shRNAs compared with those in cells transfected with the scramble control and in MC38 cells transfected with the *S100a1* ORF compared with those in cells transfected with the empty vector. **h** CUT&Tag assays showing the binding of p65 to the proximal region of the *Csf2* promoter in LCC cells transfected with the *S100a1* shRNAs compared with that in cells transfected with the scramble control and in MC38 cells transfected with the *S100a1* ORF compared with that in cells transfected with the empty vector

To further explore the molecular mechanism through which S100A1 regulates GM-CSF expression, we conducted immunoprecipitation (IP) followed by liquid chromatography-tandem mass spectrometry (LC-MS/MS) analysis in *S100a1*^OE^ MC38 cells. The S100A1 interactome was found to be enriched in ubiquitin-associated pathways, including processes such as ubiquitin-like protein peptidase activity and deubiquitinase activity (Supplementary Fig. 12), highlighting a potential regulatory role of ubiquitination in S100A1-mediated function. Among the specific candidate binding proteins (Data S4), USP7 emerged as a significant deubiquitinase (Fig. 5a). This finding indicates that USP7 may play a critical role in modulating S100A1-related signaling pathways through its deubiquitination activity. Coimmunoprecipitation (CoIP) assays verified the interaction between S100A1 and USP7 in LLC cells and *S100a1*^OE^ MC38 cells (Fig. 5b). Immunofluorescence also revealed the colocalization of S100A1 and USP7 in *S100a1*^OE^ MC38 cells (Fig. 5c). Recent studies suggest that the loss of USP7 activity leads to enhanced ubiquitination of NF-κB, which diminishes its ability to bind to the promoter, thereby reducing the expression of target genes following activation of Toll-like and TNF receptors.^29^ Notably, the GM-CSF gene (*Csf2*) expression is regulated through the interaction of the NF-κB transcription factor p65 with a proximal region of the *Csf2* promoter.^30,31^ This evidence led us to investigate whether S100A1 could regulate GM-CSF expression via USP7-mediated deubiquitination of p65. Immunoblotting assays revealed increased p65 expression in *S100a1*^KD^ tumor cells, whereas *S100a1*^OE^ reduced the protein expression of p65 (Fig. 5d). However, S100A1 did not affect the protein expression of USP7 (Fig. 5d), implying that S100A1 might affect USP7 deubiquitination activity. USP7 is a cysteine isopeptidase belonging to the USP family.^32^ This protein is composed of an N-terminal meprin and TNF receptor-associated factor homology (MATH) domain, a catalytic domain, and a C-terminal region containing five ubiquitin-like (Ubl) domains.^33^ Previous research confirmed that the C-terminal Ubl domains are essential for facilitating the interaction between USP7 and p65.^29^ CoIP experiments with various USP7 constructs (Supplementary Fig. 13a) revealed that S100A1 binds to USP7 only when the C-terminal Ubl domains are present (Fig. 5e). Neither MATH nor USP7ΔUBL showed an appreciable binding affinity for S100A1 (Fig. 5e), confirming that the C-terminal Ubl domains are sufficient for the USP7-S100A1 interaction. These findings indicate that S100A1 loss can allosterically activate USP7 by interacting with the Ubl domains, thereby enhancing its activated state. Notably, we observed that the interaction between USP7 and p65 was increased upon *S100a1*^KD^ in LLC cells, whereas *S100a1*^OE^ attenuated the interaction between USP7 and p65 in MC38 cells (Fig. 5f), indicating that the S100A1/USP7/p65 axis regulates GM-CSF expression.

Since p65 is a critical factor in the above axis, we next aimed to assess the effect of alterations in S100A1 expression on the half-life of p65. A pulse-chase experiment using cycloheximide showed that the half-life of p65 was reduced in *S100a1*^OE^ MC38 cells, whereas S100A1 ablation led to p65 stabilization in LLC cells (Supplementary Fig. 13b, c). Furthermore, the level of p65 ubiquitination was attenuated in LLC *S100a1*^KD^ cells, whereas *S100a1*^OE^ MC38 cells exhibited a higher amount of ubiquitinated p65 compared to their controls in the presence of MG132 (Fig. 5g). These findings suggest that S100A1 contributes to the ubiquitin-mediated degradation of p65 through its interaction with USP7. Moreover, both confocal microscopy and fractionation analyses demonstrated that *S100a1*^KD^ promoted the nuclear localization of p65 in LLC cells, whereas *S100a1*^OE^ induced the opposite effects in MC38 cells (Supplementary Fig. 13d–g), indicating that the level of active p65 was increased upon *S100a1*^KD^. The increased and decreased binding of p65 to the proximal region of the *Csf2* promoter in *S100a1*^KD^ LLC cells and *S100a1*^OE^ MC38 cells, respectively, was also confirmed by CUT&Tag assays (Fig. 5h). Together, these results demonstrated that S100A1 ablation affects the deubiquitination of NF-κB by interacting with the C-terminal Ubl domain of USP7, leading to increased GM-CSF expression and M1-like macrophage polarization, thus promoting T-cell-mediated antitumor immunity.

### GM-CSF priming enhances anti-PD-1 efficacy in tumors with high S100A1 expression

**Fig. 6:**
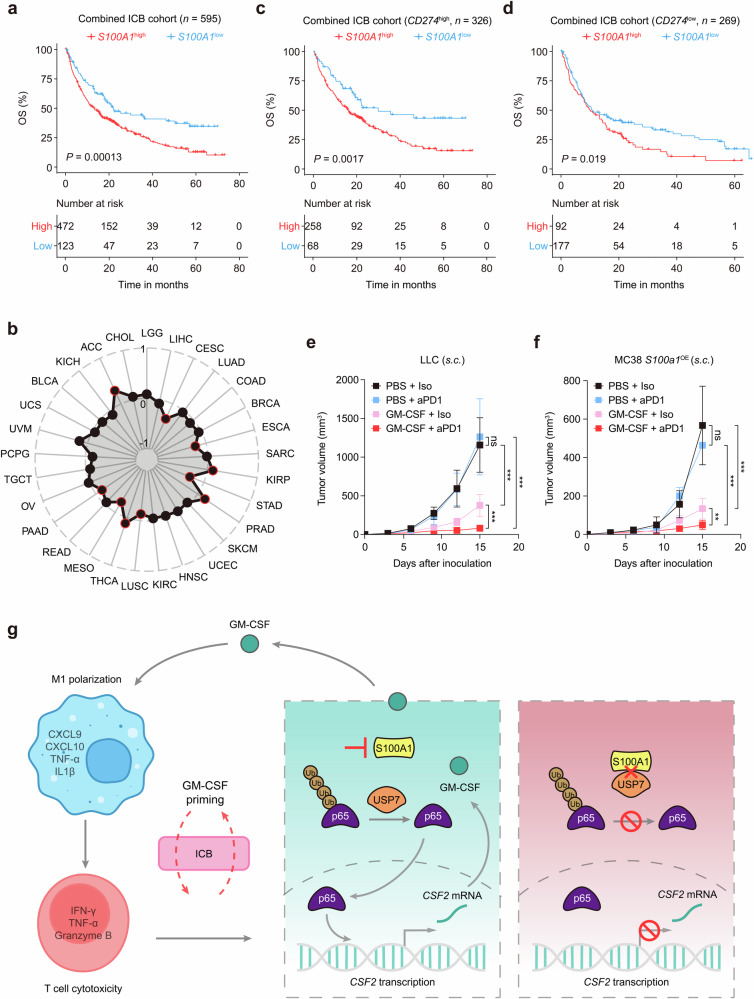
GM-CSF priming potentiates anti-PD-1 efficacy in S100A1^high^ tumor-bearing mice. **a** Kaplan‒Meier estimates of the OS between patients with high or low *S100A1* mRNA expression in the combined immunotherapy cohort. **b** Radar plot depicting the Pearson correlation between *S100A1* and *CD2*74 mRNA expression in TCGA pan-cancer cohorts. The red circle indicates a significant correlation. **c**, **d** Kaplan‒Meier estimates of the OS between the S100A1^high^ and S100A1^low^ groups of patients with high (**c**) or low (**d**) *CD2*74 mRNA expression in the combined immunotherapy cohort. **e**, **f** Tumor growth curves of control or *S100a1*^KD^ LLC tumor-bearing (**e**) and control or *S100a1*^OE^ MC38 tumor-bearing mice (**f**) receiving anti-PD-1 or isotype with or without GM-CSF priming. **g** Summary diagram describing the S100A1/USP7/p65/GM-CSF modulatory axis in regulating immune escape and potentiating immunotherapy efficacy. The schematics were generated using Adobe Illustrator (version 25.4.1)

To further validate the potential of S100A1 expression in determining immunotherapy response, we combined immunotherapy cohorts of renal cell carcinoma (*n* = 181),^34^ metastatic urothelial carcinoma (mUC; *n* = 348),^17^ skin cutaneous melanoma (*n* = 49),^14^ metastatic gastric cancer (*n* = 45),^35^ and glioblastoma (*n* = 17)^36^ patients and observed a notable difference in OS between the risk-defined groups based on S100A1 expression (Fig. 6a). Indeed, S100A1 was not a prognostic factor in various solid tumors, including NSCLC, breast cancer and melanoma (Supplementary Fig. 14a), implying its potential in predicting immunotherapy efficacy. To date, PD-L1 expression remains the most studied biomarker for predicting responses to immunotherapy in various solid tumors, such as NSCLC,^37^ breast cancer,^38^ and esophageal cancer.^39^ We found no significant correlation between *CD274* (encoding PD-L1) and *S100A1* mRNA expression in TCGA-LUAD cohort (Supplementary Fig. 14b) or the mUC anti-PD-L1 cohort^17^ (Supplementary Fig. 14c). Moreover, the pan-cancer analysis did not reveal a significant correlation between *CD274* and *S100A1* mRNA expression in multiple types of solid tumors (Fig. 6b). Notably, S100A1 retained its ability to stratify immunotherapy outcome regardless of PD-L1 expression (Fig. 6c, d), These data indicate that the effect of S100A1 on TIME remodeling and the immunotherapy response extends beyond the PD-1/PD-L1 signaling. Recent studies have shown that priming “cold” PyMT tumors with GM-CSF enhanced the responsiveness to ICB. This effect was attributed to the normalization of tumor vasculature, which reduced tumor hypoxia.^40^ These findings highlight the importance of carefully considering the context of GM-CSF when designing immunotherapy strategies. We observed that GM-CSF priming improved the anti-PD-1 sensitivity of LLC xenografts (Fig. 6e), which harbor high S100A1 expression. Similar results were obtained in *S100a1*^OE^ MC38 xenografts (Fig. 6f). These observations revealed the potential of S100A1 in predicting the ICB response and patient prognosis, as well as the role of the S100A1/USP7/p65 regulatory axis in modulating GM-CSF expression. GM-CSF priming of tumors with high S100A1 expression reversed the immunologically “cold” TME, thereby potentiating immunotherapy efficacy (Fig. 6g).

## Discussion

A common way malignant cells evade the immune response triggered by ICIs is by creating an immunologically “cold” TME characterized by tumor exclusion of cytotoxic CD8^+^ T cells.^41^ Recent efforts have been dedicated to devising approaches to “inflame” the TME, facilitating the recruitment of immune effector cells and ultimately restoring sensitivity to ICB.^42^ Tumor cells are essential in shaping the TME via paracrine and juxtacrine signaling.^6^ Beyond genetic or epigenetic alterations, tumor cell-intrinsic factors such as dysregulated signaling pathways, metabolic reprogramming, autophagy, and senescence influence the immune landscape in various solid tumors.^6^ Therefore, targeting tumor-intrinsic features offers a promising strategy to reprogram the TIME from tumor-supporting to tumor-suppressing, thus increasing the immunotherapy efficacy.

In this study, we have provided insight into the various factors that influence the ICB response in cancer. Inhibiting tumor-intrinsic S100A1 enabled macrophages and T cells to acquire antitumor properties by activating GM-CSF signaling. GM-CSF has been found to improve antigen presentation by stimulating the differentiation and activation of monocytes and DCs, while also mitigating the suppressive effects of Tregs that hinder antitumor immunity.^43,44^ Combining GM-CSF with ICB may favor T-cell-mediated antitumor responses.^45^ However, the effectiveness of GM-CSF relies on tumor-intrinsic or tumor-extrinsic factors in the TME to determine whether it fosters antitumor immunity or supports tumor growth. The contrasting roles of GM-CSF emphasize the importance of its upstream regulators in precisely manipulating the GM-CSF-mediated immune response.^46^ Our data adds to this knowledge by identifying S100A1 as a key upstream regulator of GM-CSF signaling that reprograms macrophages to an M1-like polarization state and facilitates T-cell-mediated immunity, ultimately potentiating immunotherapy efficacy. Given its immunomodulatory and proinflammatory effects, GM-CSF has been explored as a potential adjuvant in various cancer immunotherapy strategies, including the use of GM-CSF-expressing oncolytic viruses like talimogene laherparepvec, GM-CSF-secreting cancer vaccines, and the coadministration of GM-CSF with peptide antigens or antigen-loaded DCs.^47–49^ Several clinical trials have revealed an adjuvant role of GM-CSF in remodeling the immunosuppressive “cold” TME toward an inflammatory phenotype, and this effect can be further increased by ICB.^45,50^ For example, in patients with unresectable stage III or IV melanoma, the combination of ipilimumab and systemic GM-CSF (sargramostim) led to improved OS and reduced toxicity compared to ipilimumab treatment alone.^45^ The synergy observed between GM-CSF and PD-1 inhibitors in our preclinical models underscores its potential as an adjuvant to overcome resistance in immunologically “cold” tumors characterized by high S100A1 expression.

Accumulated observations have revealed the predictive potential of PD-L1 expression in immunotherapy efficacy. The KEYNOTE-024^51,52^ and KEYNOTE-042^53^ trials indicate that patients with high PD-L1 expression experience significantly greater antitumor responses and enhanced survival outcomes when treated with pembrolizumab. However, the CheckMate 227 study demonstrated that combining ICIs with chemotherapy improved survival outcomes regardless of PD-L1 levels.^54^ This suggests that PD-L1 expression may not serve as a fully reliable or independent biomarker for predicting treatment efficacy. Consequently, the choice of anti-PD-(L)1-based immunotherapy should not solely depend on the PD-L1 expression status. Interestingly, our study validates that S100A1-dependent GM-CSF signaling exerts effects on M1-like macrophage polarization and thus promotes T-cell-mediated immunity, which not only supports one mechanism by which ICB synergizes with GM-CSF but also indicates the predictive role of S100A1 as a potential biomarker to stratify patients according to the response to the combination of ICB with GM-CSF priming. Therefore, prospective studies on the translational relevance of therapies targeting S100A1 in combination with ICB are warranted.

Nevertheless, it is important to consider the following limitations of our study. Firstly, determining the plasma S100A1 levels of patients with tumor, especially those with lung cancer, is currently not as specific or definitive for immunotherapy efficacy as PD-L1, MHC, and TMB are.^55^ The relationships between plasma S100A1 expression and clinicopathological parameters have not yet been thoroughly investigated, which can present limitations in terms of consistency and reliability across diverse populations, environments, or conditions. Whether S100A1 can be used alongside other clinical data or as a standalone indicator remains unclear. Further large-scale cohort studies are warranted to identify the efficiency and robustness of S100A1 for decision-making in clinical practice. Secondly, given that S100A1 is determined to be a potential synergistic target in immunotherapy, there is a need for the development of more potent and selective S100A1 inhibitors. These inhibitors should undergo further preclinical and clinical trials to determine the effectiveness of targeting S100A1 in lung cancer and other solid tumors. Thirdly, although we revealed that the anti-PD-1 therapy induced the expression of *S100A1* in patients with breast cancer and that plasma S100A1 expression was elevated post-treatment compared with pretreatment in lung cancer patients with SD/PD, the relationship of S100A1 with the acquired resistance to ICIs needs to be analyzed. Moreover, it is essential to note that while S100A1 regulates calcium homeostasis in cardiomyocytes,^56^ this study did not investigate its role in the calcium regulation in tumor cells. The role of S100A1-associated calcium signaling in the immunotherapy response and resistance mechanism has yet to be explored. Whether the S100A1 and USP7 interaction is Ca^2+^-dependent may be important for devising S100A1-based interventions. Finally, further research is warranted to fully elucidate the signaling pathways regulated by S100A1, along with the mechanisms of its secretion and the role of post-translational modifications related to its function.

In summary, we have reported the ability of tumor-intrinsic S100A1 blockade to remodel the immunologically “cold” TME into an inflamed and immunoactive phenotype, eliciting antitumor immunity and potentiating cancer immunotherapy. Mechanistically, we identified the inhibitory effect of the S100A1/USP7/p65/GM-CSF axis on M1-like macrophage polarization and CD8^+^ T-cell reinvigoration, which contributes to immune evasion and resistance to immunotherapy. Preclinical model studies have suggested that combining ICIs with GM-CSF priming might be a potential novel immunotherapy strategy for immunologically “cold” tumors characterized by high S100A1 expression. Notably, the relevance of plasma S100A1 levels was illustrated at various levels: (1) by clustering lung cancer patients into groups with different responses to ICB treatment; (2) by establishing a potential strategy (GM-CSF as an immunostimulatory adjuvant) to prime tumor cells with high S100A1 expression to the ICB response; and (3) by revealing novel synergistic targets for cancer immunotherapy. These insights highlight the importance of understanding cancer cell-intrinsic mechanisms in advancing personalized immune-based treatments.

## Materials and methods

### Patients and specimens

The study design was approved by the ethics committee of National Cancer Center/Cancer Hospital, Chinese Academy of Medical Sciences (approval number: NCC2019C-007), and written informed consent was obtained from all patients before enrollment. To examine the correlation between plasma and tissue S100A1 expression, we collected paired blood and tissue samples from forty-four patients with lung cancer from December 2015 to February 2023, including NSCLC and SCLC. For ELISA, we collected seventy-seven pre- and post-treatment blood samples from patients with lung cancer receiving immunotherapy from December 2016 to December 2022. The diagnosis of malignancy was based on clinical criteria or histology. According to RECIST 1.1, forty-eight patients with lung cancer assessed as having PR for more than six months were defined as responders, and twenty-nine patients assessed as having SD or PD were considered nonresponders. To categorize patients into S100A1-low and S100A1-high-expression groups, we performed receiver operating characteristic (ROC) curve analysis according to PFS status. The optimal cutoff point was determined based on Youden’s Index. Patients whose S100A1 expression were below the identified cutoff were classified as the low-expression group, whereas those whose S100A1 expression were above the cutoff were classified as the high-expression group. For the IHC analysis, we collected seventy-four frozen surgically resected NSCLC tissues from patients from May 2013 to September 2018.

### Animal studies

NSG mice, BALB/c-nude mice, BALB/c mice, and C57BL/6J mice at 4–6 weeks of age were obtained from Charles River Laboratories. PtprcaRag1ko/koTg(TcraTcrb)1100Mjb/J (OT-I mice) mice were obtained from the Shanghai Model Organisms Center, Inc., for the isolation of TCR_OT1_. Mouse xenografts were generated in C57BL/6 mice via subcutaneous implantation of 1 × 10^6^ cells or intravenous injection of 1 × 10^5^ cells via the tail vein. A single-vector lentiviral, Tet-inducible shRNA system^57^ was constructed, in which the *S100a1* shRNA sequences (Supplementary Table 2) were cloned into pLKO-Tet-On vector and stably introduced into LLC cell line via lentiviral transduction. To assess the knockdown of S100A1, LLC cells were cultured in the presence of doxycycline (Dox, 2 μg/mL) for 72 hours and analyzed by immunoblot. To study pLKO-Tet-On-mediated S100A1 ablation in vivo, LLC cells stably transfected with inducible *S100a1* shRNA (Tet-on sh*S100a1* #3) or the scramble control (Tet-on Scramble) were implanted into immune-competent C57BL/6J mice and tumors were allowed to reach approximately 25–50 mm^3^, at which point Dox was administered in drinking water (ad libitum, 2 g/L) for all groups. Tumor growth was monitored every 3 d by measuring the two orthogonal external diameters using a caliper. The tumor volume (mm^3^) was calculated as (*L* × *W*^2^)/2, where L and H represent the length and width, respectively. Mice were euthanized when the maximum tumor length reached 15 mm. Tumors were excised and processed for histological and flow cytometry analysis when they reached 1000 mm^3^. All experiments with mice were performed according to protocols approved by the ethics committee of National Cancer Center/Cancer Hospital, Chinese Academy of Medical Sciences (approval number: NCC2023A516).

## Supplementary information

Supplementary material

The tissue and plasma S100A1 expression in lung cancer patients

The demographics and clinicopathological parameters of lung cancer patients receiving ICIs

The expression of S100A1, CD8, and CD86 in NSCLC tissue samples

The S100A1 interactome identified by IP-LC-MS/MS

Original immunoblots

## Data Availability

The mass spectrometry proteomics data have been deposited to the ProteomeXchange Consortium via the PRIDE^58^ partner repository with the dataset identifier PXD059275. The scRNA-seq data are available in the Gene Expression Omnibus database under accession number GSE285657.
