# Supplementary material for: Targeting tumor-intrinsic S100 calcium-binding protein A1 augments antitumor immunity and potentiates immunotherapy efficacy
Source: Signal Transduct Target Ther. 2025 Mar 17;10:99. doi: 10.1038/s41392-025-02190-2 (PMC11911448; doi:10.1038/s41392-025-02190-2)
Supplement: Supplementary file 1 — Supplementary material [file 41392_2025_2190_MOESM1_ESM.docx]

Supplementary Materials for

Targeting tumor-intrinsic S100 calcium-binding protein A1 augments antitumor immunity and potentiates immunotherapy efficacy

Yufeng Guo, Rui Wan, Jianchun Duan, Li Yuan, Zhijie Wang, Jia Zhong, Xue Zhang, Zixiao Ma, Hua Bai, Jie Wang.

Correspondence to: zlhuxi@163.com (J.W.); baihuahb@sina.com (H.B.)

**This PDF file includes:**

Materials and Methods

Figures. S1 to S14

Tables S1 to S7

Captions for Data S1 to S4

**Other Supplementary Materials for this manuscript include the following:**

Data S1 to S4 (separate file)

Materials and Methods

**Cell culture**

The syngeneic mouse lung cancer cell line LLC, the colorectal cell lines MC38 and CT26, the breast cancer cell line 4T1, the melanoma cell line B16-OVA, and the HEK293T cell line were purchased from the American Type Culture Collection. RAW264.7 cells were selected as an *in vitro* model of murine macrophages.^1^ All of these cells were cultured in Dulbecco’s modified Eagle’s medium (DMEM; Gibco) supplemented with 10% fetal bovine serum (FBS; Gibco) and 100 U/mL penicillin/streptomycin (Invitrogen). All the cell lines were verified via short tandem repeat testing. Cell culture was conducted at 37 °C in a 5% CO_2_ humidified atmosphere. All cell cultures were tested and found to be mycoplasma-free.

**BMDM isolation and *in vitro* coculture assay**

Murine BMDMs were obtained from the femurs of healthy C57BL/6J mice and matured with complete culture medium containing murine macrophage colony-stimulating factor (M-CSF, 10 ng/ml; Peprotech) for up to 7 d, with the medium refreshed every 2 days to promote differentiation. For *in vitro* coculture with tumor cells, BMDMs (1×10^6^/well) were seeded in the lower chamber of a 24 mm Transwell chamber with a 0.4 μm pore polycarbonate membrane (Corning) before coculture. Tumor cells (5 × 10^5^/well) were seeded in the upper chamber before coculture. After 24 h of incubation, the upper chamber was placed onto the lower chambers for cocultivation. BMDMs were then collected after 12 h for flow cytometry analysis or cocultured with 5 × 10^5^ preactivated TCR_OT1_ for an additional 24 h. When indicated, BMDMs were additionally differentiated in the presence of conditioned medium from LLC *S100a1*^KD^ or B16-OVA *S100a1*^OE^ cells or their isogenic controls.

**IHC staining**

Human or mouse tumor tissues were cut into 3-μm-thick sections and processed for IHC staining. The sections were incubated with rabbit polyclonal anti-S100A1, anti-CD86, and anti-CD8 antibodies diluted 1:100 overnight at 4 °C. Immunostaining was performed with the Envision System using diaminobenzidine (Dako). For the negative control, the primary antibody was replaced with PBS. The expression of S100A1, CD86, and CD8 was quantified via Image-Pro Plus software 6.0.

**Treatments**

Mouse recombinant GM-CSF (Sino Biological Inc.) was used at a concentration of 10 ng/mL. Anti-GM-CSF blocking antibody (clone B7-H1; BioXCell) or its IgG2b control isotype (clone LTF-2; BioXCell) and anti-PD-1 blocking antibody (clone RMP-14; BioXCell) with its IgG2a control isotype (clone 2A3; BioXCell) were administered intraperitoneally every 3 d at a dose of 10 mg/kg body weight. Mouse recombinant S100A1 protein (Sino Biological Inc., clone A15105B) was added to the conditioned medium at a concentration of 1 μg/mL and incubated at 37 °C for 1 h before this conditioned medium was used for functional assays.

**Gene silencing and overexpression**

Mouse *S100a1* silencing was performed via the lentiviral-driven expression of short-hairpin RNAs (**Supplementary Table 2**) based on VP001 constructs purchased from GeneChem. Nontarget short-hairpin RNA was used as a control. Lentiviral infection was performed according to the manufacturer’s protocol. Infected cells were selected via incubation with puromycin (2 μg/mL), and S100A1 downregulation/upregulation was determined by qPCR (Primer sequences listed in **Supplementary Table 3**), protein immunoblotting, and ELISA. For transient plasmid transfection, Lipofectamine RNAiMAX was used to transduce the human S100A1 ORF expression plasmid (Sino Biological Inc.) or the corresponding pCMV3-N-His vector into HEK293T cells.

**Quantification of S100A1 and GM-CSF secretion**

The cells were incubated for 24 h in DMEM supplemented with 10% FBS before collection, and S100A1 (Invitrogen) and GM-CSF (Abcam) secretion was estimated by ELISA following the manufacturer’s instructions.

***In vivo* macrophage depletion assay**

For depletion of macrophages in the model mice, the animals were pretreated for two weeks with 9 mg/kg CLD-Lp (Yeasen Biotechnology) or PBS and administered intraperitoneally every four days. Two weeks after these treatments, tumors were engrafted subcutaneously with the indicated tumor cell lines. CLD-Lp depletion was subsequently sustained, and the animals were treated with control or anti-PD-1 antibodies as previously described.

**scRNA analysis**

Mouse orthotopic models were constructed as described above. Single-cell suspensions were generated. The sequencing reads were examined by quality metrics, and transcripts were mapped to a reference mouse genome (mm10) and assigned to individual cells of origin according to the cell-specific barcodes via the Cell Ranger pipeline (10x Genomics). Transcriptomes with more than or equal to 200 total features, fewer than 4000 total features (to filter out doublets), and less than 15% of the total features derived from mitochondrial genes were retained. A total of 31,862 cells were obtained for downstream analyses, of which 15,042 cells were from the control LLC tumor, and the remaining 16,820 cells were from the *S100a1*^KD^ LLC tumor. To integrate cells into a shared space from different samples for unsupervised clustering, we used the harmony algorithm^2^ to integrate data from the control and *S100a1*^KD^ LLC tumors. The nearest neighbor graph and UMAP were created with the functions *FindNeighbors* and *RunUMAP* from the Seurat package.^3^ For gene scoring analysis, we calculated gene signatures in each cell via Seurat’s *AddModuleScore* function.

**Spatial transcriptomic analysis**

For spatial transcriptomics analysis, mouse orthotopic models were constructed as described above. After four weeks, the LLC orthotopic tumors were dissected and placed in an optimal cutting temperature (OCT)-filled mold and fresh frozen by dry ice at -40 ℃. Coronal sections of 10 mm were placed within capture areas on the Visium Spatial Gene Expression Slide. The Visium Spatial Gene Expression Slide (10 × Genomics) includes four capture areas (6.5 mm × 6.5 mm); each capture area has ~5000 gene expression spots, each spot with primers that include Illumina TruSeq Read 1 (partial read 1 sequencing primer), a 16-nucleotide (nt) spatial barcode (all primers in a specific spot share the same spatial barcode), and a 12-nt UMI; 30-nt poly(dT) sequence (captures polyadenylated mRNA for cDNA synthesis). Before a new tissue was used to generate Visium Spatial Gene Expression libraries, the permeabilization time was optimized. Briefly, the Visium Spatial Tissue Optimization workflow included placing thyroid tissue sections on seven-capture areas on a Visium Tissue Optimization slide (10 × Genomics). The sections were fixed, stained, and then permeabilized for different durations (0, 3, 6, 12, 18, 24, and 30 min). Tissue permeabilization for 30 min resulted in the maximum fluorescence signal. Next, the on-slide reverse transcription (RT) reaction was performed at 53 °C for 2 h. Second-strand synthesis was subsequently performed on the slides for 15 min at 65 °C. Library quality was assayed using a 1 μL sample on a Bioanalyzer High Sensitivity chip (Agilent). 10× Genomics Visium libraries were prepared and sequenced on Illumina NovaSeq 6000 (Illumina) in 28 bp + 120 bp paired-end sequencing mode. The sequencing data were processed via the Space Ranger pipeline (version 1.2.1; 10 × Genomics). Seurat (version 4.3.0)^3^ was used to process the Space Ranger output files. The spots with extremely low UMIs or genes were removed, and mitochondrial and ribosomal genes were filtered. After QC, we used the R package Harmony (version 1.0)^2^ to integrate the expression profiles of the control and *S100a1*^KD^ LLC tumors to perform the basic downstream analysis and visualization. Cell-type annotation was performed based on the scRNA-seq and spatial transcriptomic data via Celltrek.^4^ We performed gene set enrichment analysis via the ssGSEA algorithm in the R package GSVA^5^ to calculate enrichment scores for each cell type gene signature.

**T-cell isolation and *in vitro* coculture assay**

For T-cell isolation, TCR_OT1_ were enriched from spleens isolated from CD45.2^+^ OT-I mice using CD8 microbeads (Miltenyi Biotec) and LS columns (Miltenyi Biotec) according to the manufacturer's instructions. Isolated CD45.2^+^ TCR_OT1_ were plated onto 24 well TC-treated plates precoated with 5 μg/mL purified anti-mouse CD3 (BioLegend) and 2 μg/mL of purified anti-mouse CD28 (BioLegend) and incubated in RPMI+L-glutamine+10% FBS+50mM β-mercaptoethanol (β-ME) supplemented with 20 ng/mL recombinant murine IL-2 (Peprotech) for 24 h. For the *in vitro* coculture assay, B16-OVA cells were added (5 × 10^5^/well) to the wells of a flat-bottom TC-treated 24-well plate containing 5 × 10^5^ of preactivated TCR_OT1_ in RPMI+L-glutamine+10% FBS+50mM β-ME. After 24 h of coculture, cells were harvested, stained, and subjected to flow cytometry analysis.

**Adoptive T-cell transfer**

For adoptive T-transfer studies, 1x10^6^ TCR_OT1_ (CD45.2) were isolated as described above and adoptively transferred intravenously (i.v.) into tumor-bearing CD45.1/CD45.2 C57BL/6J mice at the indicated time points after tumor transplantation. At the indicated time points, adoptively transferred T cells were isolated from tumors and prepared for downstream flow cytometry analyses.

**Immune profiling by flow cytometry**

Implants of the indicated synergic mouse tumor cell lines were dissected from the mice at ~500 mm^3^, and the total weight was measured. Biopsies were minced using scalpels and digested with 500 U/mL collagenase IV (Sigma‒Aldrich) and 200 mg/mL DNase I (Roche) per 0.3 g of tumor weight for 30 min at 37 °C. After incubation, the cell suspensions were passed through a 70-μm cell filter to remove large pieces of undigested tissue. Erythrocytes were lysed via Red Blood Cell Lysis Solution (Solarbio). For cell surface staining, 1 × 10^6^ cells were incubated with an anti-Fc receptor blocking antibody (clone 2.4G2) and stained with the indicated antibodies in flow staining buffer for 30 min on ice. For intracellular staining, the cells were first fixed and permeabilized via a Fixation/Permeabilization Solution Kit (Cytek Biosciences). All flow cytometry analyses were performed on LSR II (BD Biosciences). Analysis of flow cytometry data was performed via FlowJo. The flow cytometry antibodies used are summarized in **Supplementary Table 4**.

**Western blot and CoIP assay**

For western blotting, proteins were extracted by lysing cultured cells in RIPA buffer containing protease inhibitors and quantified using a BCA assay kit. Later, heat-denatured proteins mixed with 1x loading buffer were separated by SDS–PAGE and then transferred onto PVDF membranes. Finally, the blots were exposed to enhanced chemiluminescence reagents using ImageQuant™ LAS 4000 after incubation with the indicated primary antibodies, followed by incubation with an HRP-conjugated secondary antibody. For the IPs, the cell lysates were incubated with the indicated antibodies at 4 °C overnight on a vertical rotator and then rotated vertically for 30 min at room temperature in the presence of protein A/G beads. Afterward, the beads were washed five times with precooled lysis buffer and mixed with SDS–PAGE loading buffer to elute proteins for immunoblotting. The sequences of primers used for constructing the USP7 deletion mutants are listed in **Supplementary Table 5**. For the ubiquitination assay, cells were transfected with the indicated DNA constructs, and cell lysates were immunoprecipitated with anti-S100A1, followed by western blotting with a primary antibody against ubiquitin. The primary antibodies used for western blot and IP assays are summarized in **Supplementary Table 6**.

**CUT&Tag assay**

The putative p65-binding sites located in the promoter region of *Csf2* were previously described.^6,7^ ChIP assays were performed using a chromatin IP kit according to the manufacturer’s protocol. Briefly, LLC *S100a1*^KD^, CT26 *S100a1*^KD^, and MC38 *S100a1*^OE^ cells, along with their isogenic control cells, were crosslinked in 1% paraformaldehyde for 10 min and then lysed with lysis buffer to extract the nuclei. After sonication, the lysate was incubated with an anti-p65 antibody (1:200) overnight at 4 °C. Protein A/G beads were used to capture the *Csf2* DNA, and qPCR was performed to amplify the DNA sample. The sequences of the primers used for the CUT&Tag assay are provided in **Supplementary Table 7**.

**LC-MS/MS analysis**

Anti-S100A1 was used for IP, and the rabbit IgG was used as control. The reaction solution (1% SDC/100 mM Tris-HCl, pH=8.5/10 mM TCEP/40 mM CAA) was added to the bead samples, which were subsequently incubated at 60 °C for 1 h to complete protein denaturation, reduction and alkylation. All the samples were analyzed on an UltiMate 3000 RSLCnano system coupled online with a Q Exactive HF mass spectrometer through a Nanospray Flex ion source (Thermo Scientific). The LC-MS/MS was carried out by Wuhan Bio-prolab Biotechnology Co., Ltd.. The raw data were analyzed via MaxQuant software (version 1.6.6) and searched against the mouse proteome sequences downloaded from Uniprot database. The enzyme digestion specificity was set to trypsin/P with a maximum of 2 missed cleavages. The peptide mass tolerances in the first search and main search were set to 20 ppm and 4.5 ppm, respectively; the fragment match tolerance was set to 20 ppm. The search results were filtered with a 1% FDR at both the peptide and protein levels. The candidate binding proteins were screened out according to the detected unique peptides and LFQ intensity. The analysis identified the specific candidate binding proteins by applying several stringent filtering criteria: (1) Proteins with LFQ intensity = 0 in the IgG group (representing the control group immunoprecipitated with nonspecific IgG) were retained to focus on specific interactions. (2) Proteins with LFQ intensity = 0 in the anti-S100A1 IP group were excluded to ensure that only detectable proteins in this group were considered. (3) A threshold of Log_2_(fold change) > 2 was applied to identify proteins with significant enrichment in the anti-S100A1 IP compared to the IgG group. (4) Additionally, proteins with > 2 unique peptides in the anti-S100A1 IP group were retained to ensure reliable identification based on peptide coverage. After these criteria were applied, 79 proteins were identified as the specific candidate binding proteins (**Data S4**), representing high-confidence interactors uniquely enriched in the anti-S100A1 IP group.

**Immunofluorescence analysis**

The cells seeded on glass coverslips were fixed with 4% paraformaldehyde for 15 min and then permeabilized with 0.1% Triton X-100 for 10 min. After being blocked with 5% fetal bovine serum for 1 h, the cells were incubated with specific primary antibodies at 4 °C overnight in a humidified box. The secondary antibodies used the next day were Alexa 488-conjugated anti-mouse IgG and 594-conjugated anti-rabbit IgG (1:1000, Invitrogen). Afterward, the nuclei were stained with hoechst for 10 min. Finally, coverslips were added to the glass slides, and the cells were viewed under a confocal laser scanning microscope (Olympus). The data were analyzed via ImageJ software.

**Statistical analysis**

GraphPad Prism 7.0.0 and R Studio were used for data analysis and visualization. The error bars represent the means ± SEMs. The student’s t-test was used to make two-way comparisons. The log-rank test was used to compare survival data. Categorical variables were analyzed by the chi-square test or Fisher’s exact test. Correlations were assessed via the Pearson correlation coefficient. The *p* values less than 0.05 were considered statistically significant.


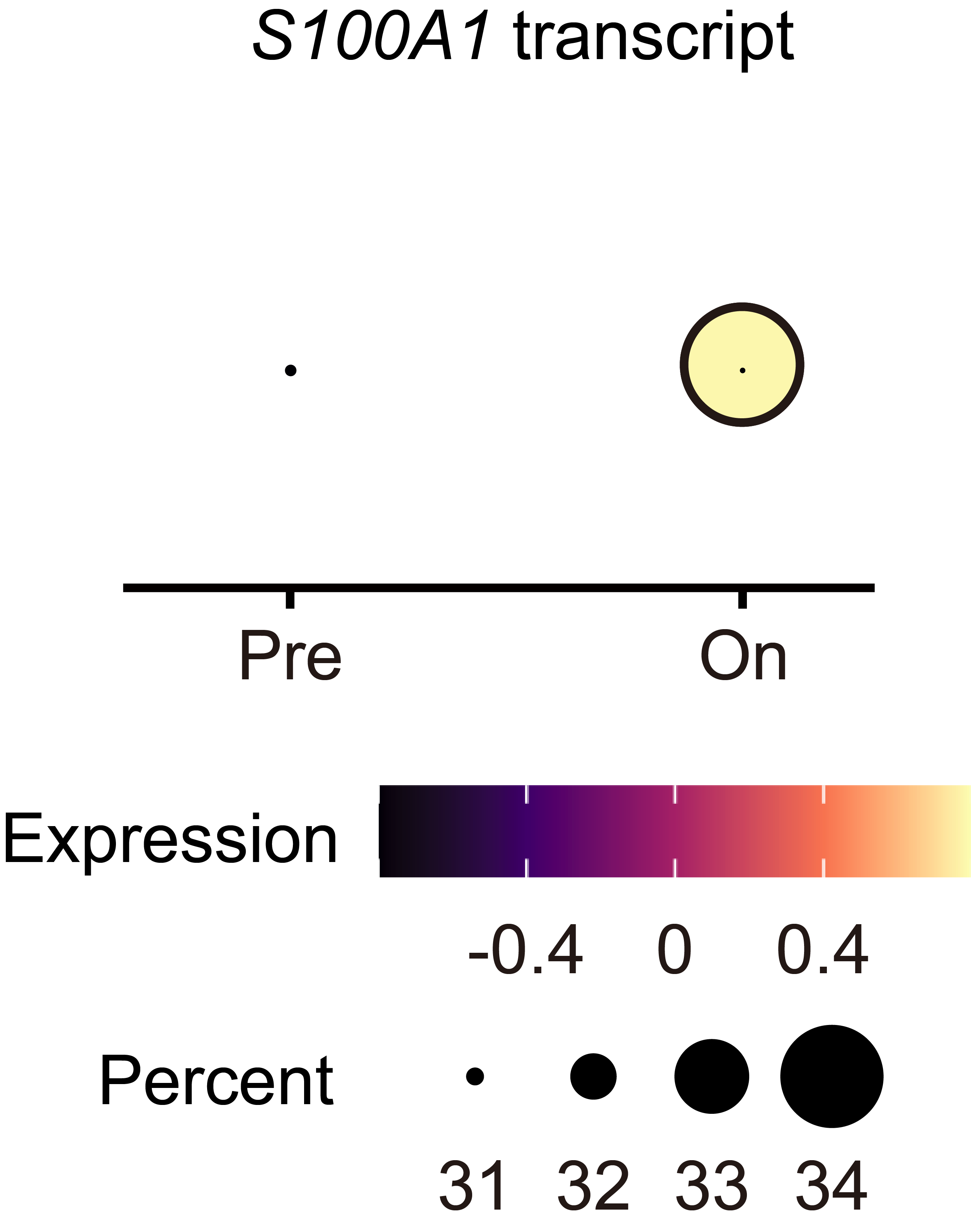


Figure. S1.

**ICI treatment induced tumor-intrinsic S100A1 expression.** Dot plot depicting *S100A1* mRNA expression of tumor cells pre- and post-treatment of anti-PD-1 based on breast cancer scRNA-seq dataset EGAS00001004809.





Figure. S2.

**The plasma S100A1 levels are positively correlated with the tissue S100A1 expression in samples from lung cancer patients. a** qPCR analysis of relative *S100A1* mRNA expression in HEK293T cells transfected with the indicated amounts of *S100A1* ORF (*S100A1*^OE^). The data are presented as the means ± SEMs (*n* = 3). **b** Immunoblot analysis of S100A1 protein expression in HEK293T cells transfected with the indicated amounts of *S100A1* ORF (*S100A1*^OE^). β-actin was used as the loading control. **c** Quantitative analysis of S100A1 ELISA in the supernatants derived from HEK293T cells transfected with the indicated amounts of *S100A1* ORF (*S100A1*^OE^). **d** Correlation analysis between the supernatant's S100A1 expression and relative *S100A1* mRNA expression in HEK293T cells transfected with the indicated amounts of *S100A1* ORF. **e** Correlation analysis between the supernatant's S100A1 and S100A1 protein expression in HEK293T cells transfected with the indicated amounts of *S100A1* ORF. **f** Representative images of the IHC analysis of S100A1 protein expression in tissue samples from lung cancer patients with high and low plasma S100A1 levels. The scale bars represent 100 μm, and the inset scale bars represent 50 μm. **g** Pearson correlation analysis of S100A1 protein expression with plasma S100A1 levels of tissue samples from lung cancer patients.


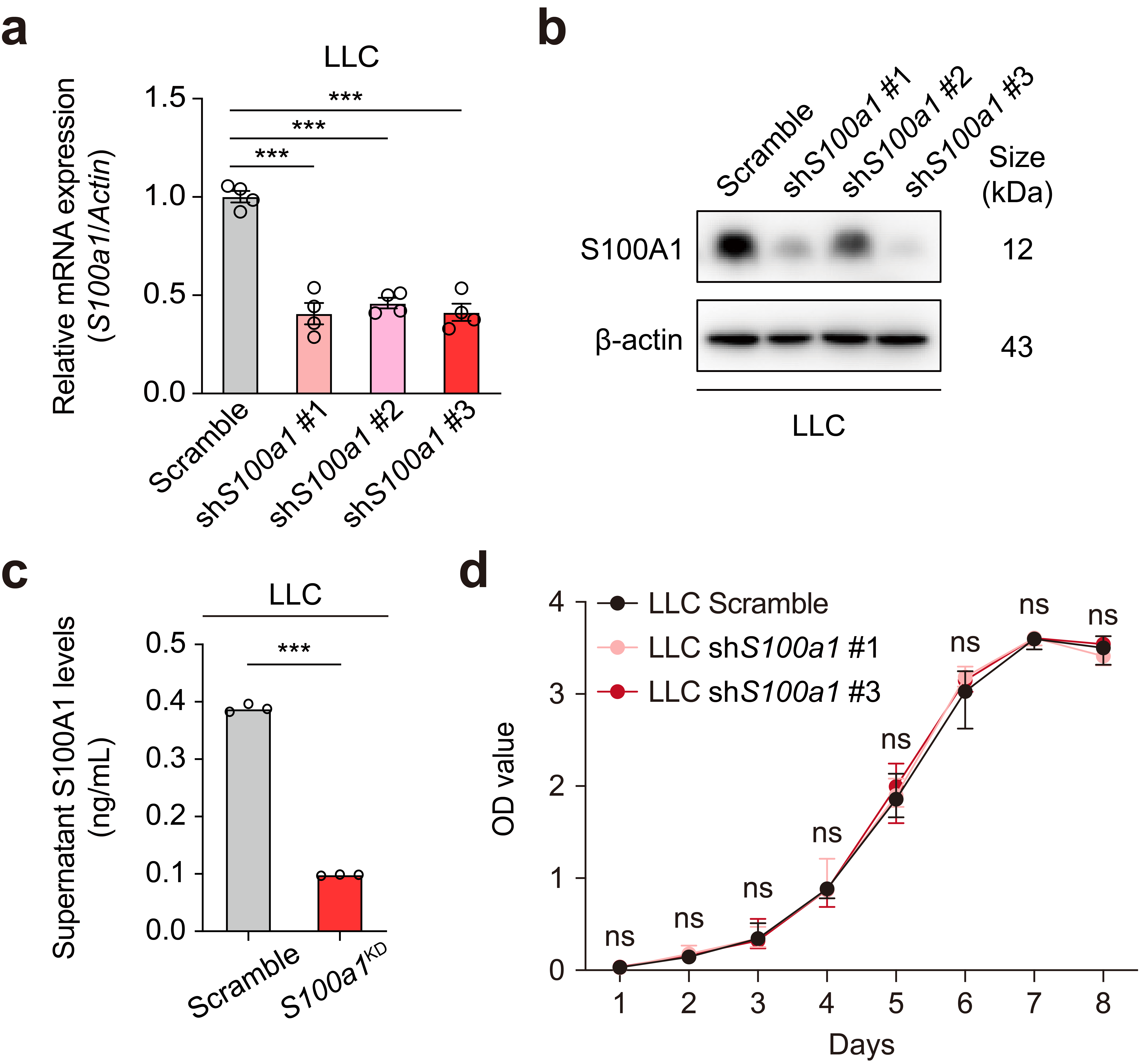


Figure. S3.

**S100A1 was not essential for cancer cell proliferation. a** qRT-PCR analysis of relative *S100A1* mRNA expression in LLC cells transfected with the *S100A1* shRNAs or the scramble control. The data are presented as the means ± SEMs (*n* = 4, ****p* < 0.001). **b** Immunoblot analysis of S100A1 protein expression in LLC cells transfected with the *S100A1* shRNAs or the scramble control. β-actin was used as the loading control. **c** Quantitative analysis of S100A1 ELISA in the supernatants derived from control and *S100a1*^KD^ LLC cells. The data are presented as the means ± SEMs (*n* = 3, ****p* < 0.001). **d** CCK8 viability assay in LLC cells transfected with the *S100A1* shRNAs or the scramble control. The data are presented as the means ± SEMs (*n* = 4, ns: not significant). OD, optical density.





Figure. S4.

**Tumor-intrinsic S100A1 loss affects tumor immune evasion. a** Tumor growth curves of control and *S100a1*^KD^ CT26 in nude mice (left panel) and BALB/c mice (right panel). The data are presented as the means ± SEMs (*n* = 6, ***p* < 0.01, ****p* < 0.001). **b** Tumor growth curves of control and *S100a1*^KD^ 4T1 in nude mice (left panel) and BALB/c mice (right panel). The data are presented as the means ± SEMs (*n* = 6, ***p* < 0.01, ****p* < 0.001). **c** Immunoblot analysis of S100A1 protein expression in LLC cells transfected with Tet-on sh*S100a1* or Tet-on Scramble incubated in the presence or absence of Dox (2 μg/mL) for 72 h. β-actin was used as the loading control. **d** Representative images of the IHC analysis of S100A1 protein expression in tissue samples from Tet-on sh*S100a1* or Tet-on Scramble LLC subcutaneous xenografts. After tumors reached approximately 25-50 mm^3^, all tumor-bearing mice were administered Dox in drinking water (ad libitum, 2 g/L). The scale bars represent 100 μm. **e** Tumor growth curves and survival analysis of tumor-bearing mice as described in **d**.





Figure. S5.

**Tumor-intrinsic S100A1 loss remodels the TIME. a** The numbers of different immune cell populations in TIME in control and *S100a1*^KD^ LLC tumors. The data are presented as the means ± SEMs (*n* = 4, ns: not significant, **p* < 0.05, ****p* < 0.001). **b** CD86^+^ and I-A/I-E^+^ macrophages in control and *S100a1*^KD^ LLC tumors determined by flow cytometry. The data are presented as the means ± SEMs (*n* = 4, ***p* < 0.01, ****p* < 0.001). **c** The numbers of CD4^+^ and CD8^+^ T cells in control and *S100a1*^KD^ LLC tumors. The data are presented as the means ± SEMs (*n* = 4, ns: not significant, ***p* < 0.01). **d** CD69^+^CD8^+^ T cells in control and *S100a1*^KD^ LLC tumors determined by flow cytometry. The data are presented as the means ± SEMs (*n* = 4, ****p* < 0.001). **e** Ki67^+^CD8^+^ T cells in control and *S100a1*^KD^ LLC tumors determined by flow cytometry. The data are presented as the means ± SEMs (*n* = 4, ****p* < 0.001). **f** PD1^+^TIM3^+^CD8^+^ T cells in control and *S100a1*^KD^ LLC tumors determined by flow cytometry. The data are presented as the means ± SEMs (*n* = 4, ns: not significant).





Figure. S6.

**Tumor-intrinsic S100A1 loss promotes M1 macrophage polarization. a** The UMAP plot of all the cell populations in the TME. **b, c** Stacked pie charts (**b**) and UMAP density plots (**c**) showing the compositions of different cell populations in the control and *S100a1*^KD^ LLC orthotopic xenografts. **d** The tSNE plot of the subclusters of macrophages in the control and *S100a1*^KD^ LLC orthotopic xenografts. **e** tSNE plots showing the expression of M2-like macrophage marker genes (*Arg1, Thbs1, Pdpn, Adam8,* and *Abca1*) in the subclusters of macrophage population. **f** tSNE plots showing the expression of M1-like macrophage marker genes (*H2.DMb1*, *H2.DMa*, *H2.Eb1*, *H2.Aa*, and *Aif1*) in the subclusters of macrophage population. **g** tSNE plots showing the expression of alveolar macrophage marker genes (*Plet1*, *Ear2*, *Krt79*, *Cidec*, and *Ear1*) in the subclusters of the macrophage population. **h** Pathway enrichment scores of phagocytosis, angiogenesis, and M1 and M2 polarization in different subclusters of macrophages in the control and *S100a1*^KD^ LLC orthotopic xenografts.


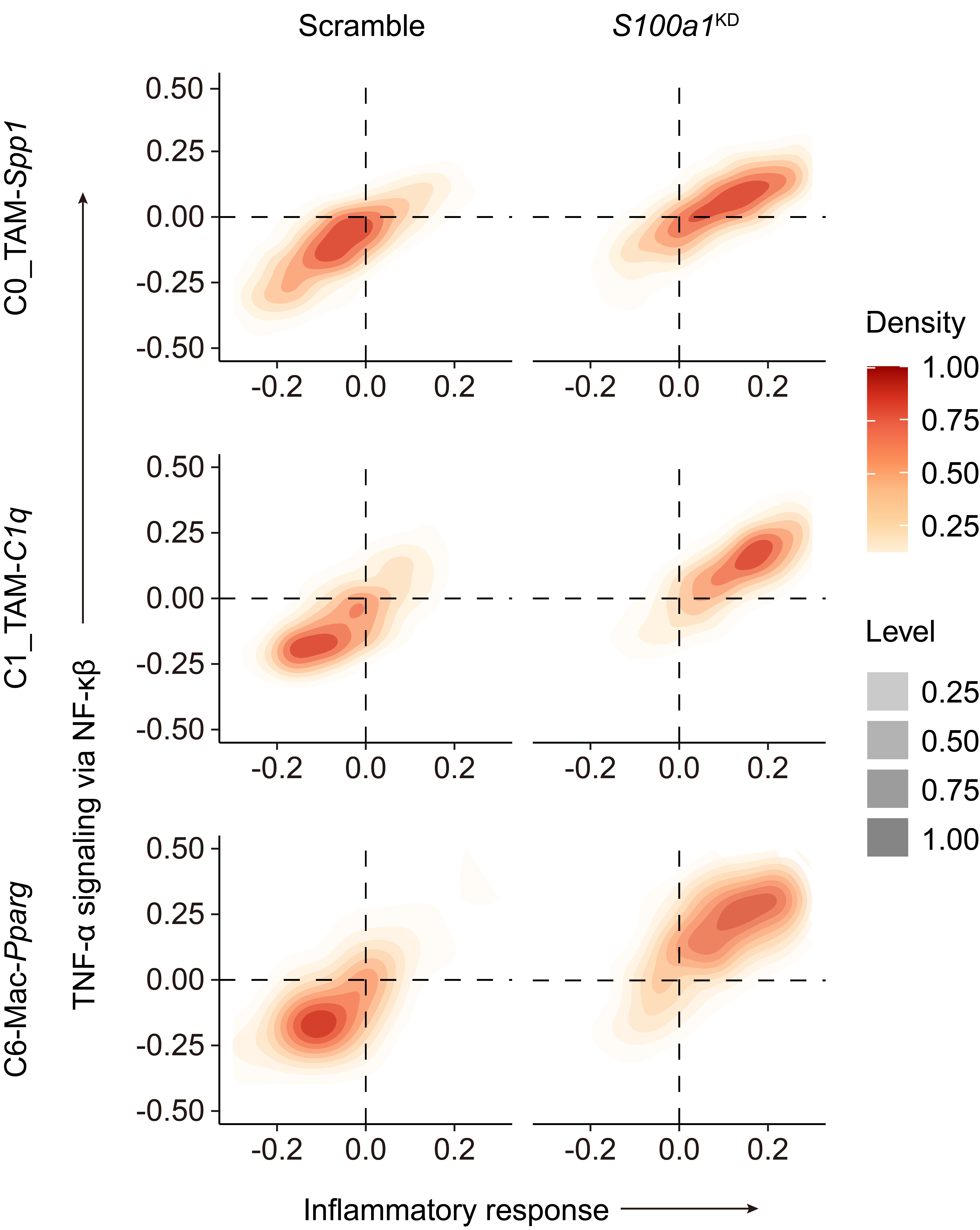


Figure. S7.

**Tumor-intrinsic S100A1 loss stimulates** **proinflammatory M1-like signaling**. Contour plots showing pathway enrichment scores of TNF-α signaling via NF-κβ and inflammatory response in the subclusters of macrophages in the control and *S100a1*^KD^ LLC orthotopic xenografts.





Figure. S8.

**Tumor-intrinsic S100A1 loss enhances T cell-mediated antitumor immunity. a** The tSNE plot of the subclusters of the CD8^+^ T cell population in the control and *S100a1*^KD^ LLC orthotopic xenografts. **b** tSNE plots showing the expression of *Tcf7* and *Lef1* in the subclusters of the CD8^+^ T cell population. **c** tSNE plots showing the expression of *Nkg7*, *Ccl5*, and *Mki67* in the subclusters of the CD8^+^ T cell population. **d** Comparisons of *Nkg7* and *Ccl5* expression in the CD8^+^ T cell population in the control and *S100a1*^KD^ LLC orthotopic xenografts. **e** PHATE plots of the subclusters of tumor-infiltrating CD8^+^ T cells in the control and *S100a1*^KD^ LLC orthotopic xenografts. **f** Contour plots showing pathway enrichment scores of T cell activation, surface receptor signaling, and cytokine production in the subclusters of the CD8^+^ T cells in the control and *S100a1*^KD^ LLC orthotopic xenografts.





Figure. S9.

**Ablation of tumor-intrinsic S100A1 potentiates an inflamed TME. a** The spatial distribution of different immune cell populations in the spatial transcriptomic data of the control and *S100a1*^KD^ LLC orthotopic xenografts. **b** The spatial distribution of macrophage M1 polarization and T cell-inflamed scores in the spatial transcriptomic data of the control and *S100a1*^KD^ LLC orthotopic xenografts.





Figure. S10.

**Tumor-intrinsic S100A1 facilitates T cell function via modulating M1 macrophage polarization. a** Gating strategy of tumor-infiltrating CD45.2^+^CD8^+^ OT-I T cells immunophenotyping: Cells were gated based on dimensions (FSC-H *vs.* SSC-H), and single-cell were gated for doublet discrimination (FSC-A *vs.* FSC-H). Live cell populations were separated, and dead cells were excluded by gating out using Live/Dead stains (FSC-A *vs.* Live/Dead). CD8^+^ single positive (CD8) and CD45.1^-^CD45.2^+^CD8^+^ OT-I T cells were gated based on the fluorescence intensity for CD8^+^, CD45.1, and CD45.2. **b** The numbers of CD45.2^+^CD8^+^ OT-I T cells in control and *S100a1*^OE^ B16-OVA tumors. The data are presented as the means ± SEMs (*n* = 4, ****p* < 0.001). **c** Flow cytometry analysis of total TCR_OT1_, IFN-γ^+^ TCR_OT1_, and TNF-α^+^ TCR_OT1_ in the TIME of the control and *S100a1*^OE^ B16-OVA tumors. **d** Flow cytometry analysis of iNOS^+^ RAW264.7 cells after *in vitro* coculture with control or *S100a1*^KD^ LLC cells. The data are presented as the means ± SEMs (*n* = 4, ****p* < 0.001).





Figure. S11.

**S100A1 regulates GM-CSF expression. a–c** qRT-PCR (**a**), immunoblot (**b**), and ELISA (**c**) of S100A1 and GM-CSF expression in LCC and CT26 cells transfected with the *S100a1* shRNAs compared with that in cells transfected with the scramble control and in MC38 cells transfected with the *S100a1* ORF compared with that in cells transfected with the empty vector. The data are presented as the means ± SEMs (*n* = 4, ****p* < 0.001).


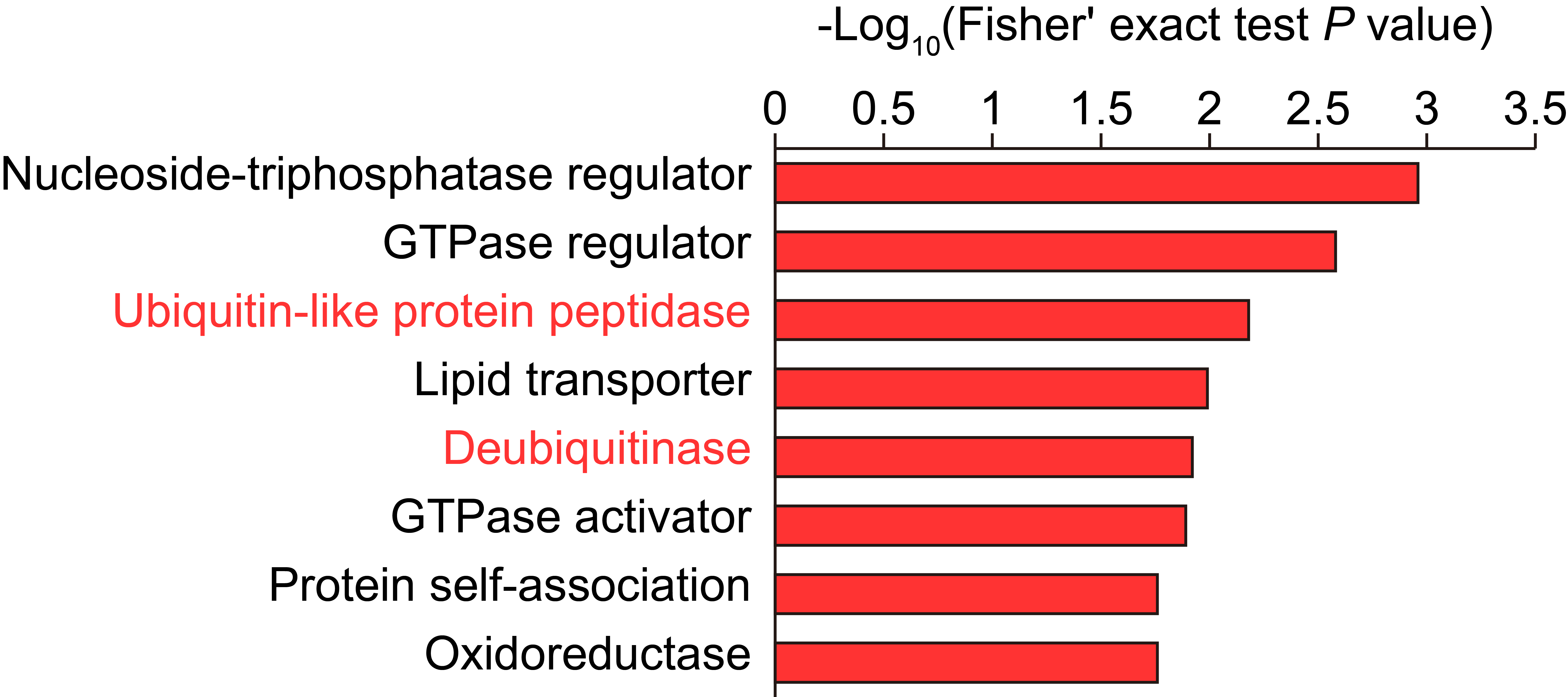


Figure. S12.

**The S100A1 interactome is enriched in ubiquitin-associated pathways.** Gene Ontology analysis of the S100A1 interactome based on LC–MS/MS.





Figure. S13.

**S100A1 affects the half-life and nuclear localization of p65. a** Various USP7 constructs for CoIP experiments. **b, c** Immunoblot analysis of the effect of S100A1 on the half-life of p65 in LLC (**b**) and MC38 (**c**) cells treated with cycloheximide (CHX) (100 mg/mL) for the indicated periods. **d, e** Immunofluorescence staining of the subcellular distribution of p65 in control and *S100a1*^KD^ LLC cells (**d**) and control and *S100a1*^OE^ MC38 cells (**e**). The scale bars represent 10 μm. Line charts of fluorescence signal positioning analysis. **f, g** Immunoblot analysis of cytoplasmic and nuclear p65 expression in LCC cells transfected with the *S100a1* shRNAs compared to that in cells transfected with the scramble control (**f**) and in MC38 cells transfected with the *S100a1* ORF compared to that in cells transfected with the empty vector (**g**). H3 and β-actin were used as the loading controls for nuclear and cytosolic fractions, respectively.





Figure. S14.

**S100A1 expression shows no significant correlation with patient prognosis and PD-L1 expression. a** Forest plot showing the hazard ratio (HR) of *S100A1* mRNA expression on the OS in TCGA pan-cancer cohort. The error bars are 95% confidence intervals. **b, c** Correlation analysis between *S100A1* and *CD274* mRNA expression in TCGA-LUAD cohort (**b**) and the mUC anti-PD-L1 cohort (**c**).

| **Characteristics** | **Plasma S100A1^high^**  **(*n* = 34)** | **Plasma S100A1^low^**  **(*n* = 43)** | ***P* value** |
| --- | --- | --- | --- |
| Age (years) | 61.0 ± 10.5 | 60.0 ± 7.0 | 0.652 |
| Gender |  |  | 0.883 |
| Female | 5 (14.7%) | 8 (18.6%) |  |
| Male | 29 (85.3%) | 35 (81.4%) |  |
| Smoking history |  |  | 0.151 |
| Never | 8 (23.5%) | 13 (30.2%) |  |
| Curent or former | 25 (73.5%) | 24 (55.8%) |  |
| N.A. | 1 (2.9%) | 6 (14.0%) |  |
| Histology |  |  | 0.333 |
| NSCLC | 24 (70.6%) | 36 (83.7%) |  |
| SCLC | 7 (20.6%) | 4 (9.3%) |  |
| Other | 3 (8.8%) | 3 (7.0%) |  |
| Stage |  |  | 0.719 |
| I | 0 (0.0%) | 1 (2.3%) |  |
| II | 1 (2.9%) | 1 (2.3%) |  |
| III | 6 (17.6%) | 5 (11.6%) |  |
| IV | 27 (79.4%) | 36 (83.7%) |  |
| Differentiation |  |  | 0.145 |
| Well | 0 (0.0%) | 2 (4.7%) |  |
| Moderate | 9 (26.5%) | 4 (9.3%) |  |
| Poor | 13 (38.2%) | 21 (48.8%) |  |
| N.A. | 12 (35.3%) | 16 (37.2%) |  |
| Driver mutation |  |  | 0.149 |
| EGFR | 1 (2.9%) | 0 (0.0%) |  |
| KRAS | 5 (14.7%) | 5 (11.6%) |  |
| Other | 1 (2.9%) | 9 (20.9%) |  |
| No | 23 (67.6%) | 26 (60.5%) |  |
| N.A. | 4 (11.8%) | 3 (7.0%) |  |
| Treatment |  |  | 0.081 |
| ICB | 5 (14.7%) | 15 (34.9%) |  |
| ICB + Chemo | 29 (85.3%) | 28 (65.1%) |  |
| Lines of treatment |  |  | 0.193 |
| Neoadjuvent | 1 (2.9%) | 0 (0.0%) |  |
| First | 29 (85.3%) | 41 (95.3%) |  |
| Second | 1 (2.9%) | 1 (2.3%) |  |
| Third | 3 (8.8%) | 0 (0.0%) |  |
| Fourth | 0 (0.0%) | 1 (2.3%) |  |
| Clinical response |  |  | 0.033 |
| PR | 16 (47.1%) | 32 (74.4%) |  |
| SD | 12 (35.3%) | 9 (20.9%) |  |
| PD | 6 (17.6%) | 2 (4.7%) |  |

Table S1. The crosstab analysis of lung cancer patients receiving ICIs based on plasma S100A1 expression

| *S100a1* shRNAs | Sequence (5’-3’) |
| --- | --- |
| shRNA-1 | FORWARD: acCCTCATCAATGTGTTCCAT  REVERSE: ATGGAACACATTGATGAGGGT |
| shRNA-2 | FORWARD: agTGGCTTGTAACAACTTCTT  REVERSE: AAGAAGTTGTTACAAGCCACT |
| shRNA-3 | FORWARD: gaTGTCCAGAAGGATGCAGAT  FORWARD: ATCTGCATCCTTCTGGACATC |

Table S2. Mouse *S100a1* shRNAs

| Target | Sequence (5’-3’) |
| --- | --- |
| *S100a1* | FORWARD: 5’-GACCCTCATCAACGTGTTCCA  REVERSE: 5’-CCACAAGCACCACATACTCCT |
| *Actin* | FORWARD: 5’-CATGTACGTTGCTATCCAGGC  REVERSE: 5’-CTCCTTAATGTCACGCACGAT |

Table S3. Primers for qPCR

| Antibody | Catalog No. | Manufacturer |
| --- | --- | --- |
| Purified Anti-Mouse CD16/CD32 | 70-0161 | Tonbo Biosciences |
| Ghost Dye^TM^ Violet 510 | 13-0870 | Tonbo Biosciences |
| Ghost Dye^TM^ Red 780 | 13-0865 | Tonbo Biosciences |
| violetFluor 450 Anti-Mouse CD45 | 75-0451 | Tonbo Biosciences |
| PerCP-Cyanine5.5 Anti-Mouse CD45 | 65-0451 | Tonbo Biosciences |
| FITC Anti-Mouse CD3e | 35-0031 | Tonbo Biosciences |
| PerCP-Cy5.5 Anti-Mouse CD8a | 65-0081 | Tonbo Biosciences |
| APC/Cyanine7 Anti-Mouse CD4 | 100414 | BioLegend |
| APC Anti-Mouse IFN gamma | 20-7311 | Tonbo Biosciences |
| PE-Cyanine7 Anti-Mouse TNF alpha | 25-7321-82 | eBioscience |
| PE Anti-Mouse Granzyme B | 12-8898-82 | eBioscience |
| FITC Anti-Human/Mouse CD11b | 35-0112 | Tonbo Biosciences |
| violetFluor 450 Anti-Mouse F4/80 | 75-4801 | Tonbo Biosciences |
| APC Anti-Mouse CD80 | 17-0801-82 | eBioscience |
| PE-Cyanine7 Anti-Mouse CD80 iNOS | 25-5920-82 | eBioscience |
| PE Anti-Mouse CD206 | 12-2061-82 | eBioscience |
| APC Anti-Mouse Ly-6G/Ly-6C (Gr-1) | 20-5931 | Tonbo Biosciences |
| APC anti-mouse CD19 | 20-0193 | Tonbo Biosciences |
| PE anti-mouse CD25 | 50-0251 | Tonbo Biosciences |
| APC anti-mouse Foxp3 | 20-0191 | Tonbo Biosciences |
| PE anti-mouse NK1.1 | 50-5941 | Tonbo Biosciences |
| APC Rat IgG2b isotype control | 20-4031 | Tonbo Biosciences |
| FITC Rat IgG2b isotype control | 35-4031 | Tonbo Biosciences |
| PE Rat IgG2b isotype control | 50-4031 | Tonbo Biosciences |
| PE-Cyanine7 Rat IgG2b isotype control | 60-4031 | Tonbo Biosciences |
| APC/Cyanine7 Rat IgG2b isotype control | 25-4031 | Tonbo Biosciences |
| violetFluor 450 Rat IgG2a Isotype Control | 75-4321 | Tonbo Biosciences |

Table S4. The antibodies used for flow cytometry

| Target | Sequence（5’-3’） |
| --- | --- |
| *USP7*-F | GGAGACCCAAGCTGGCTAGCCACCATGAACCACCAGC |
| *USP7*-R | GTTTAAACGGGCCCTCTAGATCACAAGTCCTCTTCAGAAATGAGCTTTTGCTCGTTATGGATTTTAATGGCCTTTTCAAGGTAAGTG |
| *USP7*-1-202aa-R | GTTTAAACGGGCCCTCTAGATCACAAGTCCTCTTCAGAAATGAGCTTTTGCTCTCCATGGGGAGCATCCGCCTGTAC |
| *USP7*-1-523aa-R | GTTTAAACGGGCCCTCTAGATCACAAGTCCTCTTCAGAAATGAGCTTTTGCTCTTTTGATTCCCTGATGTAGACTAACATGTAAG |
| *USP7*-202-523aa-F | GGAGACCCAAGCTGGCTAGCCACCATGGGAGTTGCGTGGGATTCAAAGAAGCAC |

Table S5. The primers for *USP7* deletion mutants

| Antibody | Catalog No. | Manufacturer |
| --- | --- | --- |
| Anti-S100A1 | ab183979 | Abcam |
| Anti-CD86 | ab220188 | Abcam |
| Anti-CD86 | 19589 | CST |
| Anti-Myc-Tag | 2276 | CST |
| Anti-USP7 | ab4080 | Abcam |
| Anti-NF-κB p65 | ab16502 | Abcam |
| Anti-iNOS | ab283655 | Abcam |
| Anti-NF-κB p65 | sc-8008 | Santa Cruz |
| Anti-USP7 | sc-137008 | Santa Cruz |
| Anti-β-actin | 8F10-G10 | zsbio |

Table S6. The antibodies used for immunoblot and IP

| Target | Seuquence (5’-3’) |
| --- | --- |
| Primer 1 | FORWARD: AAAATAGCCCCAGAGGTCCG  REVERSE: CAGCACCATGTATACCGCCA |
| Primer 2 | FORWARD: GCCTGACAACCTGGGGGAAG  REVERSE: TGATTAATGGTGACCACAGAACTC |
| Primer 3 | FORWARD: AATTCTGCAGCCACATCCTC  REVERSE: GCCAGGAGATTCCACAACTC |

Table S7. The primers for p65-binding sites within *Csf2* promoter

Data S1 to S4. (separate file)

Data S1. The tissue and plasma S100A1 expression in lung cancer patients.

Data S2. The demographics and clinicopathological parameters of lung cancer patients receiving ICIs.

Data S3. The expression of S100A1, CD8, and CD86 in NSCLC tissue samples.

Data S4. The S100A1 interactome identified by IP-LC-MS/MS.

**References**

1 Berghaus, L. J. *et al.* Innate immune responses of primary murine macrophage-lineage cells and RAW 264.7 cells to ligands of Toll-like receptors 2, 3, and 4. *Comp Immunol Microbiol Infect Dis* **33**, 443-454 (2010).

2 Korsunsky, I. *et al.* Fast, sensitive and accurate integration of single-cell data with Harmony. *Nat Methods* **16**, 1289-1296 (2019).

3 Hao, Y. *et al.* Integrated analysis of multimodal single-cell data. *Cell* **184**, 3573-3587 e3529 (2021).

4 Wei, R. *et al.* Spatial charting of single-cell transcriptomes in tissues. *Nat Biotechnol* **40**, 1190-1199 (2022).

5 Hanzelmann, S., Castelo, R. & Guinney, J. GSVA: gene set variation analysis for microarray and RNA-seq data. *BMC Bioinformatics* **14**, 7 (2013).

6 Sasaki, C. Y., Ghosh, P. & Longo, D. L. Recruitment of RelB to the Csf2 promoter enhances RelA-mediated transcription of granulocyte-macrophage colony-stimulating factor. *J Biol Chem* **286**, 1093-1102 (2011).

7 Sasaki, C. Y., Slemenda, C. F., Ghosh, P., Barberi, T. J. & Longo, D. L. Traf1 induction and protection from tumor necrosis factor by nuclear factor-kappaB p65 is independent of serine 536 phosphorylation. *Cancer Res* **67**, 11218-11225 (2007).
